# A SARS-CoV-2 variant-induced NTD-targeting antibody enhances viral infection via a distinctive binding mode

**DOI:** 10.1371/journal.ppat.1013828

**Published:** 2026-02-10

**Authors:** Wenting Li, Congcong Liu, Yaning Li, Qi Gui, Lin Cheng, Qing Fan, Bing Zhou, Haiyan Wang, Xiangyang Ge, Zheng Zhang, Renhong Yan, Bin Ju

**Affiliations:** 1 Institute for Hepatology, National Clinical Research Center for Infectious Disease, Shenzhen Third People’s Hospital, The Second Affiliated Hospital, School of Medicine, Southern University of Science and Technology, Shenzhen, Guangdong Province, China; 2 Department of Biochemistry, Key University Laboratory of Metabolism and Health of Guangdong, SUSTech Homeostatic Medicine Institute, School of Medicine, Institute for Biological Electron Microscopy, Southern University of Science and Technology, Shenzhen, Guangdong Province, China; 3 Tsinghua-Peking Joint Center for Life Sciences, School of Life Sciences, Tsinghua University, Beijing, China; 4 Department of Infectious Diseases, Affiliated Hospital of Southwest Medical University, Luzhou, Sichuan Province, China; 5 Guangdong Key Laboratory for Anti-infection Drug Quality Evaluation, Shenzhen, Guangdong Province, China; 6 Shenzhen Research Center for Communicable Disease Diagnosis and Treatment, Chinese Academy of Medical Sciences, Shenzhen, Guangdong Province, China; Institut Pasteur, FRANCE

## Abstract

SARS-CoV-2 infection elicits both neutralizing and non-neutralizing monoclonal antibodies (mAbs), primarily targeting to the N-terminal domain (NTD), receptor-binding domain (RBD), and S2 subunit of the spike protein. Notably, a unique subset of NTD-targeting mAbs isolated from prototype Wuhan-Hu-1 strain infected donors displayed a capacity of facilitating the viral infection independent of the fragment crystallizable (Fc) region *in vitro*. However, the rapid evolution of SARS-CoV-2 variants, particularly with NTD mutations, has led to widespread immune evasion. Whether SARS-CoV-2 variants could still induce NTD-targeting infection-enhancing antibodies (NIEAs) remains unclear. Here, we identified a distinctive NIEA, ConD-854, from a Delta variant primarily infected donor, with broad infection-enhancing activities against most pre-Omicron variants but not against post-Omicron variants. Structural and functional analysis revealed that ConD-854 enhanced the viral infection through an Fc-independent bivalent binding mechanism with a largely shared recognition epitope, but its heavy-light chain orientation was nearly perpendicular relative to the reported prototype strain-induced NIEAs. Collectively, our findings demonstrated that the primary infection of Delta variant could still induce the NIEAs targeting the similar epitope as those elicited by prototype strain infection. Mutations in Delta NTD were located outside the infection-enhancing epitope and did not affect the induction of NIEAs. Remarkably, we defined a distinctive structural paradigm for an NIEA to recognize the viral epitope. These results enriched our understanding of antiviral antibodies and provided insights for future vaccine design.

## Author summary

SARS-CoV-2 infection triggers the production of various antibodies, however, in *in-vitro* assays, some antibodies targeting the N-terminal domain (NTD) of the viral spike protein unexpectedly promote viral entry into cells in an Fc-independent manner. Previously, it remained unclear whether infection with SARS-CoV-2 variants such as Delta would still trigger the body to produce such NTD-targeting infection-enhancing antibodies (NIEAs). In this study, we identified one NIEA from a donor primarily infected with the Delta variant, which enhanced the infection of most pre-Omicron viruses but did not enhance the infection of Omicron and subsequent variants. Our cryo-electron microscopy (cryo-EM) data revealed that this Delta-induced NIEA, like NIEAs induced by the prototype strain, binds to a roughly similar region on the NTD containing the Loop211–215 but exhibits a distinct binding mode when compared with the previously reported prototype-induced NIEAs, as its heavy-light chain orientation is nearly perpendicular to that of the prototype-induced NIEAs even though their approaching angles are similar. Our study provides new insights into the functional mechanism of NIEAs and the design of vaccines.

## Introduction

The COVID-19 pandemic, caused by the infection of severe acute respiratory syndrome coronavirus 2 (SARS-CoV-2), has led to significant global morbidity and mortality, as well as widespread social and economic disruption, since its emergence in late 2019 [1,2]. The entry of SARS-CoV-2 into host cells is mediated by its surface spike glycoprotein, composed of S1 and S2 subunits [3]. The S1 subunit includes a receptor-binding domain (RBD), which mediates its recognition to the host cell receptor angiotensin-converting enzyme 2 (ACE2), and an N-terminal domain (NTD). While the precise function of the NTD remains incompletely understood, it is thought to play roles in viral attachment, conformational change, and immune evasion [2,4–9]. The S2 subunit is responsible for viral fusion and entry. The RBD is immunodominant and could elicit abundant antibodies, serving as a primary target of neutralizing antibodies (nAbs) [10]. There is evidence that other regions of the spike, particularly the NTD, also significantly contribute to antigenicity [11,12]. However, the neutralizing activity of NTD-targeting nAbs is generally weaker than that of RBD-targeting nAbs [12–14]. Given its role in the antigenicity and immune evasion, the antibody response to the NTD of emerging SARS-CoV-2 variants should be carefully considered in the design of the spike protein-based COVID-19 vaccines.

The NTD contains multiple glycosylation sites within its five highly variable loop structures as N1 (residues 14–26), N2 (residues 67–79), N3 (residues 141–156), N4 (residues 177–186), and N5 (residues 246–260) [11]. In contrast to the RBD, most of anti-NTD nAbs target a convergent antigenic supersite comprising the N1, N3, and N5 loops, as well as several other epitopes [11,15–19]. Additionally, some non-neutralizing NTD-targeting infection-enhancing antibodies (NIEAs) *in vitro* have been identified in individuals infected with the prototype strain of SARS-CoV-2 [11,18,20–23]. These NIEAs bind to a specific NTD epitope facing to the viral membrane, located outside the neutralization supersite and adjacent to the NTD variable loops [11,18]. Several key amino acid residues, including W64, H66, K187, V213, and R214, mediate this interaction, as alanine substitution mutants significantly reduce the binding of most of NIEAs[22]. The infection-enhancing mechanism involves bivalent antibody cross-linking of two NTDs from adjacent spikes, inducing an open RBD state to enhance SARS-CoV-2 binding to ACE2 [22,24]. While these NIEAs alone could enhance the infectivity of SARS-CoV-2 *in vitro*, this effect is obscured in the presence of high levels of nAbs [18].

The continuous emergence of SARS-CoV-2 variants accompany with immune evasion capabilities has posed a persistent threat to public health and challenged the efficacy of current therapeutic antibodies and vaccines [25–28]. Compared to other regions of the spike protein, the NTD exhibits the highest diversity with a multitude of substitutions and deletions [29]. Most NTD mutations found in SARS-CoV-2 variants are concentrated in the peripheral region of the spike, near the supersite, likely facilitating the viral escape from nAbs and fine-tuning the viral entry efficiency [29,30].

The Delta variant harbored key NTD mutations, including T19R, G142D, E156del, F157del, and R158G, clustered in the antigenic supersite which directly results in disruption of the antigenic supersite, reducing neutralization of antibodies targeting this region. Among them, G142D directly impaired the binding of NTD-specific nAbs by altering surface charge and steric hindrance, contributing to partial immune escape from convalescent or vaccine-induced sera [17,31,32]. Moreover, this mutation was retained in Omicron and most of its subvariants including BA.2.86. E156/F157 deletion and R158G reshape the 143–154 loop and further destabilized the supersite, enhancing evasion [33,34], however, they were absent in major later variants. T19R mutation disrupts the Asn-linked glycosylation motif, resulting in loss of the 17Asn-18Leu-19Thr glycan [35]. Of note, while the T19R mutation was not conserved in later variants, the T19I substitution emerged independently in certain Omicron subvariants (e.g., BA.2, BA.4/5, XBB, and BA.2.86), indicating ongoing selective pressure at this site [31,36–38]. To date, all previously reported NIEAs were isolated from individuals infected with the SARS-CoV-2 prototype strain, some of which maintain activity against early variants [11,18,20,21,23]. However, whether primary infection of SARS-CoV-2 variants could also elicit NIEAs remains undetermined.

In this study, we reported an NIEA, ConD-854, isolated from an individual primarily infected with Delta variant, exhibiting potent infection-enhancing capability to most pre-Omicron variants *in vitro* in an entirely FcγR independent way. Based on the antibody escape mutant analysis and cryo-EM structural analysis, we characterized the recognition epitope of ConD-854, which is global similar but local distinctive with that of prototype-induced NIEAs, as the heavy-light chain orientation of the former is markedly different. This study provided the first evidence demonstrating that the SARS-CoV-2 Delta variant primary infection was able to elicit NIEAs specifically targeting a previously reported NTD region and revealed another distinctive heavy-light chain binding mode of NIEAs.

## Results

### Identification of an NIEA from an individual primarily infected with SARS-CoV-2 Delta variant

**Fig 1:**
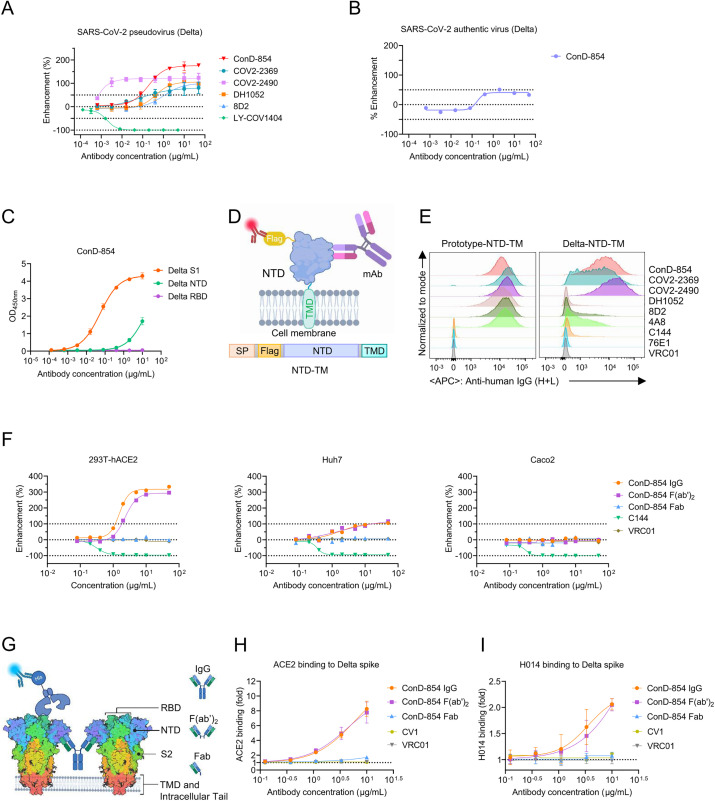
Characterizing the *in vitro* potency of an NIEA, ConD-854, isolated from a Delta variant primarily infected donor. **(A)** Pseudovirus-based evaluation of infection-enhancing activities by ConD-854 and previously reported prototype-induced NIEAs (COV2-2369, COV2-2490, DH1052, and 8D2) against the SARS-CoV-2 Delta variant. LY-COV1404, a neutralizing RBD-targeting antibody, was used as a control. **(B)** Authentic SARS-CoV-2 Delta virus infection enhancement by ConD-854 in Vero E6 cells. **(C)** Binding assay determined by ELISA evaluating the binding capability of ConD-854 to S1, NTD, and RBD of SARS-CoV-2 Delta spike. mAbs were tested at an initial concentration of 10 μg/mL and serially diluted by 5-fold. **(D)** Flag-NTD-TM expression plasmid design for analyzing NTD-targeting antibody specificity. The transmembrane region (TM) sequence is from PDGFRβ gene. **(E)** Staining of ConD-854 and prototype-induced NIEAs (10 μg/mL) on the NTD-TM expressed on the surface of HEK293T transfected cells. The prototype-NTD-specific nAb 4A8, RBD-specific nAb C144, and S2-specific nAb 76E1 are used as positive and negative control here respectively. **(F)** Pseudovirus-enhancement curve of full-length IgG-, F(ab’)_2_- and Fab-form ConD-854 for Delta variant, tested in HEK293T-hACE2 (left), Huh7 (middle) and Caco2 (right) cells. **(G)** A cartoon model of recombinant ACE2 protein binding capability detection to whole spike in the present of full-length IgG-, F(ab’)_2_- and Fab-form ConD-854 determined by flow cytometry. Detection of antibody concentration-dependent enhancement of ACE2 **(H)** and H014 **(I)** binding to the cell-surface expressed SARS-CoV-2 Delta variant spike by IgG-, F(ab’)_2_- and Fab-form ConD-854. Binding capacity was quantified as relative MFI, calculated by the fold change in MFI of GFP-positive cells bound to variant-specific spike proteins for each mAb. CV1 is a nAb targeting SARS-CoV-2 NTD, and VRC01 is an HIV-1 specific mAb, both of which were used as negative controls here. (A) The enhancement rate are calculated from three independent experiments and represented in mean values ± standard deviation (SD). (B), (E), and (F) are representative of at least two independent experiments. The OD_450nm_ values in (C) are from three independent experiments and presented as mean ± SD. The data in (H) and (I) are from at least three independent experiments and presented as mean ± SD. (D) and (G) were generated with BioRender (biorender.com).

In our previous study characterizing antibodies induced by SARS-CoV-2 Delta variant infection, we performed single memory B cell sorting using the Delta S1 subunit as a probe to isolate specific mAbs from an individual primarily infected with Delta variant [39]. Nine Delta-S1 positive mAbs were isolated, among which six bound to Delta-RBD, whereas the other three did not. Subsequent screening for neutralizing activity against SARS-CoV-2 revealed that ConD-854 did not inhibit viral infection but instead enhanced the infectivity of Delta variant to target cells *in vitro* (Fig 1A). Notably, ConD-854 exhibited a higher maximum enhancing efficacy against the Delta variant than all previously reported NIEAs (COV2–2369, COV2–2490, DH1052, and 8D2) derived from individuals primarily infected with the prototype strain [18,20], despite being less potent than COV2–2490 (Fig 1A). In addition, ConD-854 could also enhance the infectivity of authentic SARS-CoV-2 Delta variant in Vero E6 cells (Fig 1B). The infection-enhancing efficacy of ConD-854 for the Delta pseudovirus was also observed in Vero E6 cells, but was moderately lower than that in 293T-ACE2 cells (S1A Fig). Enzyme-linked immunosorbent assay (ELISA) analysis confirmed that, like COV2–2490, ConD-854 specifically bound to Delta S1 and NTD proteins but not RBD (Figs 1C and S1B). To further evaluate the binding capacity of ConD-854 to the NTD under physiological conditions, Flag-tagged NTD subunits fused with the transmembrane domain of PDGFRβ were transiently transfected into HEK293T cells (Fig 1D). Flow cytometry analysis revealed strong binding of ConD-854 to cell-surface NTDs, with comparable intensity to several prototype-induced NIEAs (Fig 1E). These results indicated that primary infection of Delta variant can indeed induce NIEAs like ConD-854.

### Mechanism of ConD-854 enhancing the infectivity of SARS-CoV-2

Previous studies have shown that bivalent binding of NIEAs to two NTDs of inter-spike could induce an open RBD conformation and then reinforce the spike binding to ACE2 [21–24]. To investigate the mechanism of in-vitro infection-enhancement by ConD-854, we measured infection-enhancing activities of IgG-, F(ab’)_2_-, and Fab-form ConD-854 to SARS-CoV-2 and their capabilities to enhance spike protein binding to ACE2. F(ab’)_2_- and Fab-form ConD-854 were obtained through different enzymatic digestions and subsequent purification, with their purity and integrity verified by SDS-PAGE and flow cytometry (S1C and S1D Fig). While retaining binding activity to spike as confirmed by ELISA (S1E Fig), the monovalent Fab format lost infection-enhancing activity. By contrast, F(ab’)_2_-form ConD-854 markedly increased the entry of Delta variant into HEK293T-hACE2 cells with comparable activities of IgG-form ConD-854, suggesting that the bivalent binding is essential for its infectivity-enhancing activity (Fig 1F). We also observed that the efficacy of ConD-854 to enhance the infection of Delta variant was attenuated in Huh7 cells, which express ACE2 at lower levels in an endogenous manner compared to HEK293T-hACE2 cells with exogenously overexpressed ACE2 (Fig 1F). Furthermore, little to no infection enhancement by IgG-, F(ab’)_2_-, and Fab- forms of ConD-854 was observed in Caco-2 cells (Fig 1F), a gastrointestinal tract cell line that has been shown to express considerably lower ACE2 levels than HEK293T-ACE2 cells [21]. The infection-enhancing efficacy of ConD-854 was in alignment with ACE2 expression levels, suggesting infectivity-enhancing activity of ConD-854 was ACE2 dependent.

Furthermore, we analyzed the effect of ConD-854 on the augmentation of ACE2 binding by detecting the binding level of soluble ACE2 protein with His tag to the spike-expressed HEK293T cells when incubating with three formats of ConD-854 at different concentrations (Figs 1H and S2). Furthermore, control antibodies (CV1 and C135) tested separately did not enhance soluble ACE2 binding to membrane spike proteins above the baseline level set by the unrelated mAb VRC01 (S2C Fig). CV1 and C135 are nAbs isolated from donors infected with the prototype strain, targeting the NTD and RBD of the SARS-CoV-2 spike, respectively [40,41]. Both antibodies, which are cross-reactive against the D614G and Delta spike variants in a flow cytometry-based binding assay (S2B Fig), but do not promote ACE2 binding (S2C Fig), were therefore included as negative controls. VRC01, an HIV-1-specific mAb [42], served as an unrelated negative control to represent baseline ACE2 binding in the absence of specific interference (S2B and S2C Fig). These results confirmed that ConD-854 facilitated SARS-CoV-2 infection by promoting ACE2 binding to an open-state RBD, triggered by the bivalent antibody cross-linking of NTDs, consistent with previously reported mechanisms [21–24]. Distinct from most reported anti-RBD neutralizing antibodies such as C135 (Class 3) and C144 (Class 1/2) that recognize epitopes accessible in both open and closed RBDs, the nAb H014 recognizes a cryptic epitope that is only exposed when the RBD is in the open state [43]. Prototype-induced NIEAs enhance the binding of H014 to the cell-surface spike protein, but do not enhance the binding of C135 and C144 [22]. Consistent with previous findings [22], our data demonstrate that the IgG and F(ab’)_2_ formats of ConD-854 significantly enhanced H014 binding (Fig 1I). The Fab fragment of ConD-854, however, showed no such enhancing effect on H014 binding. Additionally, no enhancement of H014 binding was observed with the negative controls, CV1 or VRC01. These results confirm that ConD-854 displays the typical phenotypic and functional properties of an NIEA.

To investigate the interplay between the NIEA ConD-854 and nAbs, we assessed the overall neutralization efficacy of their combinations. As shown in S3A and S3B Fig, pre-mixing ConD-854 with nAbs H014 or C144 at fixed ratios (1:1 and 10:1) and then serially diluting the entire mixture resulted in a significant reduction of the overall neutralization potency compared to the nAbs alone. This dampening effect on the mixture’s overall neutralizing efficacy was particularly pronounced for the combination with H014, a nAb with relatively moderate intrinsic neutralization activity. A pronounced overall infection-enhancing effect was observed at the intermediate dilutions of the antibody serial dilution series, specifically when the ratio of ConD-854 to H014 was 10:1. In contrast, the combination with the more potent nAb C144 was less affected by interference from ConD-854. While a slight decrease in the mixture’s overall neutralization was still detectable at the 10:1 ratio, the effect was negligible at the 1:1 ratio.

Similarly, when ConD-854 was held at different fixed concentrations while nAbs were serially diluted, the overall neutralization efficacy of the solutions was significantly lower than that of the nAb alone groups (S3C Fig). This was especially obvious at lower nAb concentrations, where the inherent enhancing effect of ConD-854 became dominant, leading to a net enhancement of infection. This phenomenon was more prominent in mixtures containing H014 compared to those with C144 (S3C Fig). Of note, under the experimental conditions tested, we did not observe a significant increase in the overall neutralization potency for any combination, including ConD-854 with H014, thus cannot directly demonstrate that the NIEA ConD-854 can boost the neutralizing activity of H014 which targets the cryptic epitopes of the spike protein by promoting its binding to the open-state spike. These observations only reflect the overall neutralizing effect resulting from the combination of a nAb and an NIEA.

### Infection-enhancing activity of ConD-854 to different SARS-CoV-2 variants

**Fig 2:**
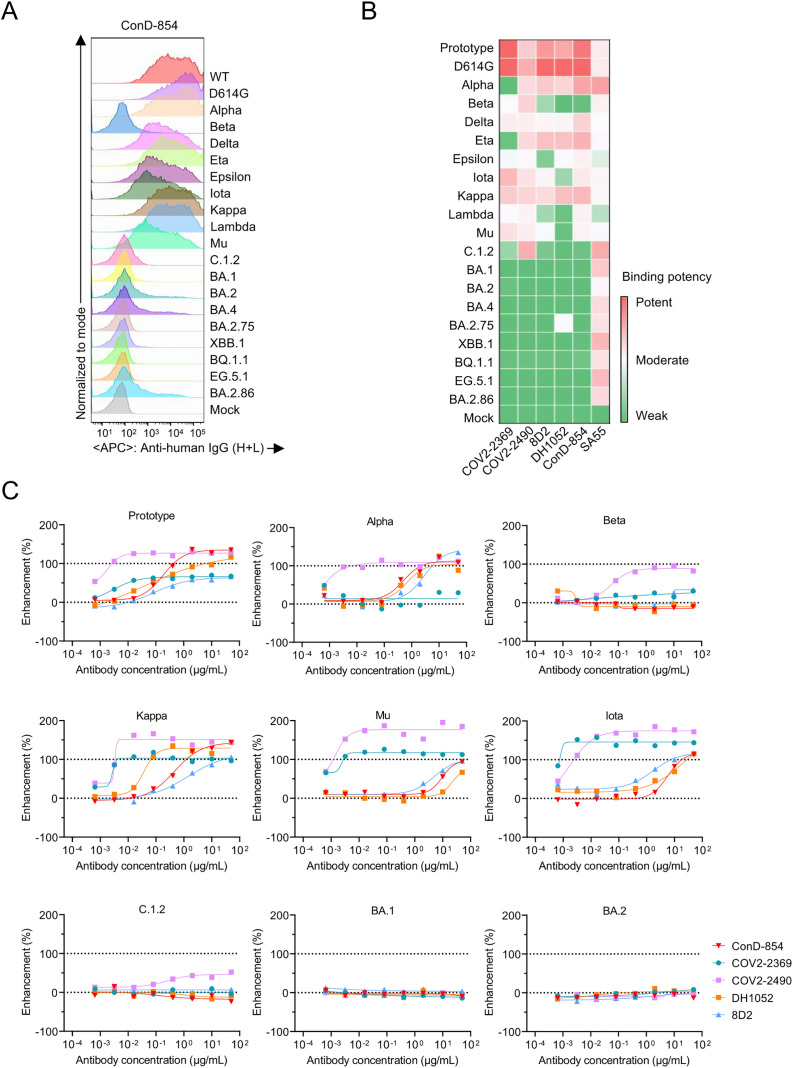
The binding and functional activity of ConD-854 to SARS-CoV-2 variants. **(A)** ConD-854 bound to the cell-surface spike of different SARS-CoV-2 variants. **(B)** Binding capacity was quantified as relative MFI, calculated by the fold change in MFI of GFP-positive cells bound to variant-specific spike proteins for each mAb, normalized against the VRC01 control. The RBD-targeting broad-spectrum nAb SA55 is used as a positive control. **(C)** Pseudovirus-based assay for estimating the cross-reactive infection-enhancing activity of NIEAs against multiple SARS-CoV-2 variants. The representative data of two independent experiments are shown here.

Most NTD-targeting nAbs recognize a common region on NTD and can be easily escaped by the SARS-CoV-2 variants [44–46]. To assess the cross-reactivity of ConD-854, we measured its binding abilities to the full-length spike proteins of various SARS-CoV-2 variants expressed on the surface of transfected HEK293T cells, using prototype-induced NIEAs as controls. The results revealed that ConD-854 efficiently bound to the spikes of most pre-Omicron variants, such as Alpha, Delta, and Lambda, but failed to recognize various Omicron variants including BA.2.86 (Fig 2A and 2B). Although emerging before Omicron, Beta and C.1.2 variants could not be recognized by ConD-854 as determined in the flow cytometry assay, which is consistent with the results of ELISA (S4 Fig). Using pseudovirus-based infection enhancement assay, we further evaluated the breadth of infection-enhancing activity of ConD-854 to several representative SARS-CoV-2 variants. As shown in Fig 2C, the infection-enhancing activity of ConD-854 was only observed in several pre-Omicron variants, such as Alpha, Kappa, Mu, and Iota, but not in post-Omicron variants such as BA.1 and BA.2, compatible with its binding ability to their spike proteins (Figs 2A, 2B, and S4). A similar correlation was also observed for previously reported prototype-induced NIEAs. These results demonstrated that ConD-854 displayed a limited cross-reactivity of enhancing infection against SARS-CoV-2 variants due to the loss of its binding capacity to mutated spike proteins.

### Key amino acid residues in NTD affecting the recognition of ConD-854

**Fig 3:**
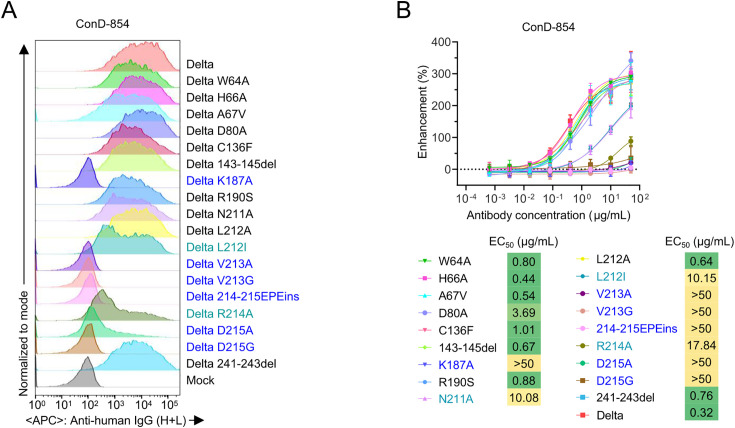
Identification of the epitope on the NTD recognized by ConD-854 using site-directed mutagenesis. **(A)** ConD-854 binding to full-length spike mutants on transfected cells, comprising single-point mutations from S5A Fig and previously reported alanine substitutions (W64, H66, K187, V213, R214) in the NTD (known as infection-enhancing sites), was analyzed by flow cytometry. Light blue-highlighted mutations indicate reduced ConD-854 binding, while dark blue-highlighted mutations indicate nearly abolished binding to cell surface-expressed Delta spike. Representative data from two independent experiments are shown. **(B)** Assessment of ConD-854-mediated infection-enhancement against SARS-CoV-2 Delta single-point mutant pseudoviruses (from A). The EC_50_ corresponding to the infection enhancement activity of Delta single-point mutant strains are shown below. Notably, when the EC_50_ value exceeds 50 μg/mL, the mutant strain exhibits no infection enhancement effect. The data in (B) are from three independent experiments and presented as mean ± SD.

Possible mutations in the NTD of spike protein conferring escape from ConD-854 were inferred by analyzing its binding to different spike variants on the surface of transfected cells and the sequence alignment (S5A Fig). Based on the distinct infection-enhancing activities of ConD-854 against SARS-CoV-2 variants, a series of amino acid mutations were introduced into Delta spike, including potential escape mutations from ConD-854 (such as L212I (BA.1 variant), V213G (BA.2 variant), D215G (Beta and C.1.2 variants)) and several alanine substitutions at key NTD residues (W64, H66, K187, V213, and R214) previously involved in NIEAs binding [22]. Using this panel of mutated spike proteins and pseudoviruses, we assessed the binding ability and infection-enhancing activity of ConD-854 *in vitro*. As shown in Fig 3A, the binding capacities, determined by mean fluorescence intensity (MFI), of ConD-854 to cell-surface Delta spike proteins were markedly reduced in L212I, and R214A mutants, and completely lost in K187A, V213A, V213G, D215A, D215G substitution mutants, as well as in the 214–215EPE insertion mutant. In the viral infection enhancement experiment, ConD-854 lost its infection-enhancing activity against Delta pseudoviruses carrying single-point substitution K187A, V213A, V213G, R214A, D215A, D215G or 214–215EPE insertion mutations, respectively, showing a significant reduction when mAb concentration = 50 μg/mL, and EC_50_ > 50 μg/mL for all these mutants except R214A (EC_50_ = 17.84 μg/mL). Furthermore, its activity was markedly diminished against pseudovirus harboring N211A, or L212I mutations showed no statistically significant reduction when mAb concentration = 50 μg/mL, but the EC_50_ for both still increased substantially (to approximately 10 μg/mL) (Figs 3B and S5B). These findings demonstrated that residues 187 and 211–215, which are known to influence the binding of prototype-induced NIEAs[21, 22], also critically affected the binding and enhancing activity of ConD-854 to SARS-CoV-2.

### Structural basis of ConD-854 binding to SARS-CoV-2 Delta NTD

To determine the epitope recognized by ConD-854, we first performed the competitive ELISA to assess its competition with three NIEAs thus far reported with available structure information. As shown in S6 Fig, COV2–2490 exhibited the strongest competition with ConD-854 (92.6%) for binding to prototype spike trimers under the setting competitor concentration of 10 μg/mL. DH1052 and 8D2 also markedly competed with ConD-854, albeit to a lesser extent (67.9% and 67.7%, respectively, at 10 μg/mL). These results suggested that ConD-854 recognized a common or highly similar epitope binding region as previously reported prototype-induced NIEAs.

**Fig 4:**
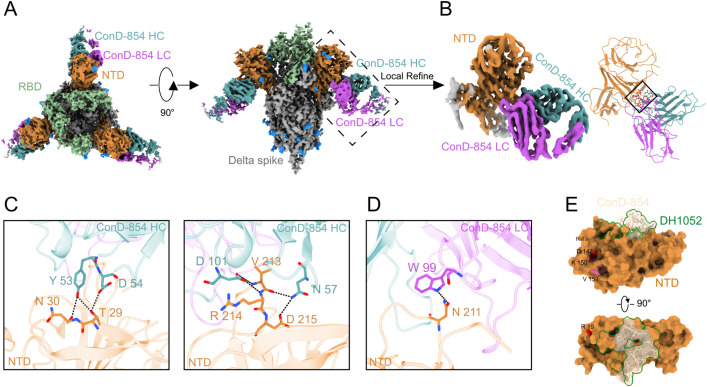
Cryo-EM structural analysis of SARS-CoV-2 Delta variant spike in complex with ConD-854 Fab. **(A)** Top (left panel) and side (right panel) views of domain-colored cryo-EM structures of ConD-854 Fab in complex with the SARS-CoV-2 Delta variant spike. **(B)** Expanded view of the interface between the NTD and ConD-854 Fab. **(C, D)** Interface contact residues between the NTD and the heavy chain (C) or light chain (D) of ConD-854 Fab. **(E)** Footprint of ConD-854 on NTD is shown as a transparency surface and stick model. The footprint of DH1052 (PDB: 7LAB) is outlined by a green curve. The T19R and G142D mutations are highlighted in red. The atomic models are missing between K150 and V159 (shown in pink), with E156del, F157del, and R158G located between these two residues. The NTD, RBD and other region of this spike protomer are colored orange, green, and gray, respectively. Glycans are colored blue. The heavy chain of ConD-854 Fab is colored dark cyan, and light chain is colored magenta.

To investigate the molecular interactions between ConD-854 and the spike protein, we first prepared the complex of ConD-854 IgG with the spike protein of the SARS-CoV-2 Delta variant for sample preparation and cryo-EM data acquisition. For subsequent structural analysis, we exclusively selected the density corresponding to the spike trimer and the Fab fragment of ConD-854 for calculation. Ultimately, the cryo-EM structure of this spike-Fab variable region complex was determined at an overall resolution of 3.4 Å. (Figs 4A, S7 and S8, and S1 Table). The spike trimer displays an asymmetric conformation, where one of three RBDs adopts the “up” conformation and the other two RBDs are in the “down” conformation. The spike-ConD-854 complex contains three ConD-854 Fabs that bind to the three NTDs of the spike protein (Fig 4A). To improve the quality of the density map in the NTD-ConD-854 interaction region, symmetry expansion and focused refinement were performed, yielding a map with a resolution of 3.4 Å. This enabled a detailed analysis of their interactions (Fig 4B). The binding of ConD-854 Fab to the Delta spike trimer is primarily mediated by complementary determining regions (CDRs) H2, H3, and L3, as determined by ImMunoGeneTics (IMGT) (Fig 4C and 4D). Specifically, Y53 and D54, which belong to CDRH2, can form three hydrogen bonds with T29 and N30 on the NTD (Fig 4C). Notably, the main chain nitrogen and oxygen atoms of V213 on the NTD form hydrogen bonds with D101 on CDRH3 and N57 on CDRH2, and the main chain nitrogen atom of R214 stabilize the interaction through hydrogen bonding with D101on CDRH3. Additional stabilization occurs via side chain of D215 hydrogen bonding with N57. Additionally, contact of the ConD-854 light chain to Delta spike trimer occurs between N211 on the NTD and W99 on CDRL3 by forming a hydrogen bond (Fig 4D). Loop211–215 on the NTD can interact with both the heavy and light chains of ConD-854, playing an important role in the binding of ConD-854 to the NTD, which is consistent with the role of the key amino acid residues that affect the recognition of ConD-854 as shown in Fig 3. A comparison of the epitope footprints on NTD surface for ConD-854 and DH1052 (PDB: 7LAB), a prototype-induced NIEA [18], showed that their epitope regions are largely overlapping (Fig 4E). Moreover, the mutations in the Delta variant (T19R, G142D, E156del, F157del, and R158G) are located outside the binding epitope of ConD-854. In total, ConD-854 binds to the known infection-enhancing site on the NTD, which does not include any mutations present in Delta variant.

### Distinctive structural paradigm for ConD-854 compared to other NIEAs

**Fig 5:**
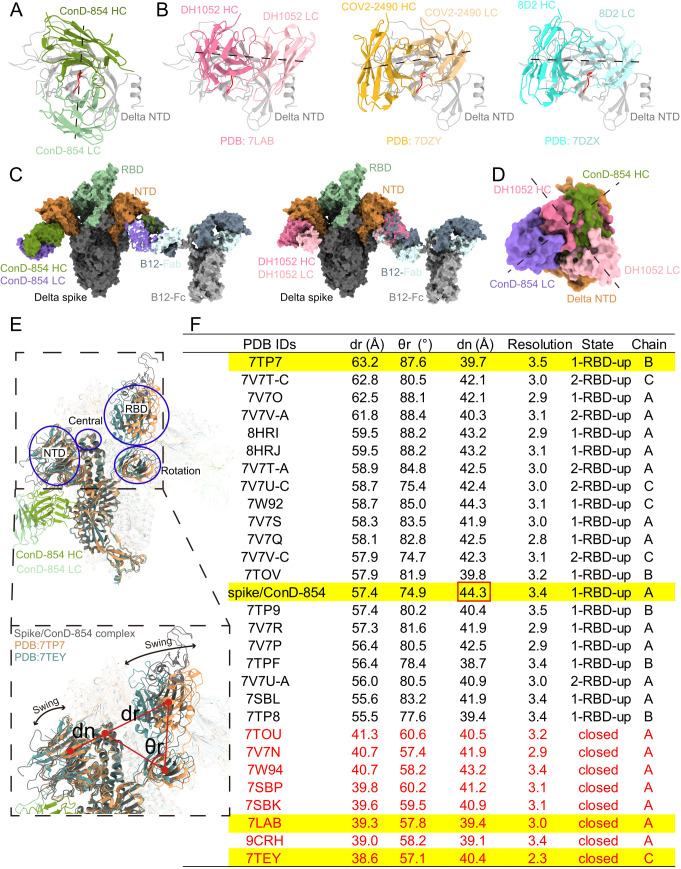
Structural basis of SARS-CoV-2 Delta spike recognition by ConD-854. **(A, B)** Top-down views of the Delta NTD superimposition for ConD-854 (A) and three prototype-induced NIEAs: DH1052 (PDB: 7LAB), COV2-2490 (PDB: 7DZY), and 8D2 (PDB: 7DZX) (B), colored by green, pink, orange, and blue, respectively. The NTDs are colored gray, and the Loop211-215 (involved in NIEAs binding) is highlighted in red. **(C)** Atomic models of Delta NTD in complex with ConD-854 Fab (left) and DH1052 Fab (right) are docked onto a full-length B12 IgG[47] (PDB: 1HZH), respectively. **(D)** The orientation of ConD-854 on the Delta NTD is rotated about 90° clockwise as relative to DH1052. The NTD, RBD, and other region of spike protomer are colored orange, green, and dark gray, respectively. Heavy chain of ConD-854 is colored dark green and light chain is colored purple. Heavy chain of DH1052 is colored dark pink and light chain is colored light pink. The heavy chain, light chain, and Fc of B12 are shown in slate blue, light cyan, and gray, respectively. The axes connecting centroids of the heavy-light chains of Fabs are shown as black dash lines. **(E)** The angle (*θ*r) and distance (dr) to quantify RBD movement and the distance (dn) to evaluate NTD movement. Atomic models for Delta spike (PDB: 7TP7 and 7TEY) were colored orange and teal. Solid red circles represent the centroids of RBD, NTD, rotation center, and central axis. **(F)** Statistics on θr, dr, and dn in the structural analysis results of different Delta spikes.

The epitope of ConD-854 on the NTD, which faces to the viral membrane, is similar to those of NIEAs induced by prototype stain including DH1052, COV2–2490, and 8D2 [18,22] (Fig 4A). The angles of approach for ConD-854 and the three other NIEAs that bind to the NTD are similar (S9 Fig). However, after superimposing different NIEAs onto the NTD respectively and analyzing the heavy-light chain orientations in the interface, although ConD-854 targets the globally similar infection-enhancing site, which contains Loop211–215, as DH1052, COV2–2490, and 8D2, it engages this epitope in a distinctive manner (Fig 5A and 5B). Observed from a fixed view of antibody-NTD binding interface, the binding orientation of ConD-854 to the NTD is rotated by approximately 90° clockwise as relative to the binding of the other three NIEAs, revealing a novel binding mode. From another perspective, Loop211–215 lies at the interface center and is parallel to the ConD-854’s heavy-light chain centroid connecting line, but nearly perpendicular to those of the other three NIEAs. Although ConD-854 exhibits a distinct heavy-light chain binding orientation compared to the other three NIEAs, the presence of the flexible hinge between Fc and Fab in IgG ensures that NIEAs can bind to the NTD with different heavy-light chain orientations [47]. ConD-854 utilizes IGHV3–30 gene with a 15-amino-acid CDRH3 and IGKV2–30 gene with a 10-amino-acid CDRL3, which differs from the other three NIEAs (S2 Table). This phenomenon indicates that different NIEAs from distinct germline gene lineages can bind to the same epitope by different heavy-light chain configurations approaching to the antigen, enhancing our understanding of NIEAs diversity (Fig 5C and 5D).

To illustrate the conformational changes of the Delta spike in the presence or absence of binding to ConD-854, the atomic models of the Delta spike that does not bind to any other antibodies or ACE2 were downloaded from the Protein Data Bank (PDB) database. Three metrics were defined [48], including (1) the RBD angle (*θ*r), which describes the upward and downward tilting poses of the RBD and any conformations in between; (2) the RBD distance (dr), which describes the distance between the RBD and the central axis; (3) the NTD distance (dn), which describes the distance between the NTD and the central axis (Fig 5E). Through the analysis of these three metrics, the results showed that the RBD and NTD of the Delta spike are in a swinging state (Fig 5F). The dr (57.4 Å) and θr (74.9°) values of the spike bound to ConD-854 fall within the middle range of the analyzed spikes: (38.6 Å-63.2 Å) and (57.1°-88.4°), indicating that even though the NTD is bound to ConD-854, the RBD remains in a swinging state. Interestingly, the dn value of the spike bound to ConD-854 is 44.3 Å, which is the largest among the analyzed spikes, whereas the dn value of the prototype strain spike bound to the NIEA DH1052 is 39.4 Å (Fig 5F). This may be because the present study used full-length IgG for structural analysis, while the prototype strain spike-DH1052 complex analysis used Fab [18], raising the possibility that bivalent binding may play a role in pulling the NTD outward during infection-enhancing binding, reducing the wedge effect of the NTD on the RBD and facilitating the RBD to adopt the “up” state [22].

## Discussion

The SARS‐CoV‐2 spike NTD is a hotspot of genetic diversity, with a large number of functional mutations emerging in this region across variants [49]. Elucidating the antigenicity of NTD in different variants and the characteristics of NTD-inducing antibodies is pivotal for assessing the impact of NTD mutations on viral fitness and guiding vaccine design [50]. The SARS-CoV-2 Delta variant carries multiple mutations in the NTD and RBD regions of the spike protein, enhancing its ability to evade nAbs [27]. In the Delta NTD region, five mutations (T19R, G142D, E156del, F157del, and R158G) are present. These mutations are not adjacent to the known infection-enhancing epitope, which primarily contains the Loop211–215. Consequently, they did not affect the binding of most NIEAs derived from patients with primary infection by the prototype strain. However, it remains unclear whether the primary infection of Delta variant could induce the NIEAs.

In this study, we isolated and characterized an NIEA named ConD-854 from a donor who was primarily infected with Delta variant. ConD-854 targets the known infection-enhancing NTD region of spike protein. Our data demonstrate that the infection-enhancing efficacy of ConD-854 is ACE2-dependent, as shown by the markedly stronger enhancement in ACE2-overexpressing 293T-ACE2 cells compared to cells with endogenous ACE2 expression. This is consistent with the weak effects observed in Vero E6 cells across both authentic and pseudovirus systems. The low maximum enhancement rate (~50%) in the authentic virus assay further supports the role of ACE2 expression levels in influencing the enhancement magnitude. Additionally, the observed discrepancy between assay systems should consider the different quantification methods (luciferase activity versus focus forming units [FFU] counting), in addition to ACE2 expression levels, as these methodological differences complicate direct cross-assay comparisons. Furthermore, bivalent IgG or F(ab’)_2_ formats of ConD-854, but not the monovalent Fab format, facilitated the spike-ACE2 binding. Comprehensive phenotypic and functional analysis indicated that ConD-854 functioned as a canonical NIEA that augments infection through a similar underlying mechanism that crosslinking NTDs of adjacent spikes to stabilize RBDs in more open conformations, thereby promoting multivalent ACE2 engagement on host cell surfaces [22].

Notably, the role of NIEAs in mediating antibody-dependent enhancement (ADE) of viral infection *in vivo* remains controversial. Higher levels of serum NIEAs are observed in severe COVID-19 patients, suggesting some correlation between NIEAs and disease severity [22]. Interestingly, DH1052, which mediates FcγR-independent infectivity enhancement *in vitro*, protected mice and cynomolgus macaques from severe disease through FcγR-dependent effector functions, including antibody-dependent cellular cytotoxicity (ADCC) and antibody-dependent cellular phagocytosis (ADCP) [18,51]. The Fc-silenced mutant, DH1052 LALA-PG, lost its protective effect but did not increase infectious virus titers compared to the isotype control in mice, indicating that DH1052 did not enhance infection even in the absence of Fc-mediated antiviral functions [51]. Thus, the contribution of NIEAs to COVID-19 disease progression *in vivo* remains complex.

Emerging Omicron subvariants carry additional mutations within or around the core region of the infection-enhancing epitope [21]. These mutations not only enable evasion of most pre-existing NIEAs by disrupting binding but may also reduce the likelihood of inducing specific NIEAs response due to altered immunogenicity. Our data showed that ConD-854 lost binding ability to SARS-CoV-2 Beta, C.1.2, Omicron BA.1, BA.2, and other Omicron subvariants, primarily due to various mutations presented among sites of 211–215 in NTD region. The demonstration of ConD-854 as an NIEA implies that the SARS-CoV-2 Delta variant was able to elicit such antibody subset, indicating no significant change in the immunogenicity of this region.

Moreover, both our study and previous research indicate that almost all NIEAs lost functional activity to BA.1, BA.2, and other Omicron subvariants [21,23]. Notably, multiple mutations in the NTD infection-enhancing epitope of BA.1 (e.g., N211 deletion, L212I substitution, and 214–215EPE insertions) likely alter the immunogenicity of this region. Similarly, BA.2 and its subvariants carry A27S and V213G/E mutations, which may also impact the binding of NIEAs. It would be valuable to investigate whether Omicron and its subvariants, particularly BA.1, with altered NTD antigenicity in Loop211–215, are able to induce strains-specific NIEAs.

Although ConD-854 exhibits a distinctive heavy-light chain orientation when binding to the NTD compared to other NIEAs, the inherent flexibility of the IgG hinge region allows rotational freedom, enabling the full-length IgG functionality. This structural plasticity indicates that NIEAs can bind to antigens through different heavy-light chain configurations, enhancing our understanding of NIEAs recognition mode diversity.

According to our study, structural characterization of SARS-CoV-2 NIEAs thus far reveals that their binding is constrained to at least two distinct heavy-light chain orientations with limited approach angles at a concentrated epitope. By contrast, nAbs targeting NTD antigenic supersite display greater heavy-light chain orientations diversity but maintaining restricted approach angles [16]. This epitope-dependent binding behavior was also observed in other viral systems. HIV-1 CD4 binding site antibodies shared highly similar orientations, both in terms of overall antibody angle of approach and heavy-light chain orientation relative to the spike [52]. However, antibodies against other supersites of HIV-1 vulnerability, such as the membrane-proximal external region on gp41 display divergent binding orientations [53–55]. In addition, antibodies with diverse angles of approach and different heavy-light chain orientations towards a conserved lateral patch of H7N9 hemagglutinin head has been demonstrated in influenza study [56]. Thus, antibody binding plasticity is jointly shaped by target site structural features including accessibility and conservation as well as antibody’s own flexibility, particularly in its hinge region. This balance between target site constraints and antibody flexibility explains how our immune system can produce diverse effective antibody responses against viruses.

## Limitations of the study

While our *in-vitro* study provides detailed mechanistic insights into NIEA ConD-854, its actual effects in a complex living system remain unclear. The role of NIEA ConD-854 in animal models and its impact on overall infection dynamics remain essential questions for future studies. It is also noteworthy that NIEAs identified so far, including ConD-854, target a roughly similar epitope that is prone to mutational escape [11,18,20,21,23]. Concerning the natural evolution of viral escape, nearly all reported such NIEAs lose their infection-enhancing activity against post-Omicron variants. Therefore, a critical question is whether the host can generate broad-spectrum NIEAs against diverse variants, and the conditions and mechanisms underlying their production warrant further investigation. Furthermore, there is rare structural evidence to elucidate the mechanism by which NIEAs binding to this known epitope induce conformational changes to the RBD open state. Additionally, it remains unclear whether other, yet undiscovered infection-enhancing epitopes exist. Together, these aspects represent major unresolved challenges in the field that also define the inherent limitations of our current study.

## Materials and methods

### Ethics statement

This study was approved by the Ethics Committee of Shenzhen Third People’s Hospital, China (approval number 2021-030). Written informed consents have been obtained from the participant for both sample collection and subsequent analysis.

### Biological samples

The peripheral blood mononuclear cell (PBMC) sample was obtained from a patient primarily infected with the SARS-CoV-2 Delta variant. Prior to sample collection, the patient had not received COVID-19 vaccination or antibody therapy. The isolated PBMCs were preserved in freezing medium and stored in liquid nitrogen at the BioBank of Shenzhen Third People’s Hospital. Written informed consent was provided by the participant for both sample collection and subsequent analysis. The isolation process of mAbs had been reported in our previous study [39]. Here, we further investigate and describe the function and feature of an NIEA, named ConD-854.

### Antibody expression and purification

HEK293F cells were co-transfected with the antibody heavy chain and light chain expressing plasmids. Transfection was facilitated using 1 mg/mL polyethylenimine (PEI) and the cells were cultured with shaking at 125 rpm under 8% CO_2_. After six days, cell culture medium was harvested by centrifugation at 3500 × g for 30 min at 4°C, then the antibody was then purified from the supernatant using protein A affinity chromatography.

### Protein expression and purification

The extracellular domain (ECD) (1–1208 a.a.) of spike protein of SARS-CoV-2 Delta variant was cloned into the pCAG vector (Invitrogen) with six proline substitutions at residues 817, 892, 899, 942, 986 and 987 and a C-terminal T4 fibritin trimerization motif followed by 10 × His tag, respectively. A “GSAS” mutation at residues 682–685 was introduced into ECD to prevent the host furin protease digestion. The mutants were generated with a standard two-step PCR-based strategy. The plasmids used to transfect cells were prepared by GoldHi EndoFree Plasmid Maxi Kit (CWBIO).

The recombinant protein was overexpressed using the HEK293F mammalian cells at 37°C under 5% CO_2_ in a Multitron-Pro shaker (Infors, 130 rpm). When the cell density reached 2.0 × 10^6^ cells/mL, the plasmid was transiently transfected into the cells. To transfect one liter of cell culture, about 1.5 mg of the plasmid was premixed with 3 mg of polyethylenimines (PEIs) (Polysciences) in 50 mL of fresh medium for 15 min before adding to cell culture. Culture supernatant was collected by centrifugation at 4000 × g for 15 min after sixty hours transfection.

The secreted ECD was purified by Ni-NTA affinity resin (Qiagen). The nickel resin loaded was rinsed with the wash buffer containing 25 mM HEPES (pH 7.0), 150 mM NaCl and 30 mM imidazole. Protein was eluted by wash buffer plus 270 mM imidazole. Then the Ni-NTA eluent of ECD was subjected to size-exclusion chromatography (Superose 6 Increase 10/300 GL, GE Healthcare) in buffer containing 25 mM HEPES (pH 7.0), 150 mM NaCl. The peak fractions were collected and incubated with ConD-854 at a molar ratio of about 1:3.6 for one hour to assemble spike-ConD-854 complex. To remove excessive ConD-854, the mixture was subjected to size-exclusion chromatography (Superose 6 Increase 10/300 GL, GE Healthcare) in buffer containing 25 mM HEPES (pH 7.0), 150 mM NaCl. The peak fractions containing protein complex were collected for EM analysis.

### Production of pseudoviruses

HEK293T with the appropriate density were co-transfected with SARS-CoV-2 variant spike expression plasmid and Env-deficient HIV-1 backbone vector (pNL4-3.Luc.R-E). Two days after transfection, the supernatant was collected, clarified by centrifuged and filtered and stored at −80°C. Detailed sequence information of spike proteins used in this study is listed below.

**SARS-CoV-2 prototype (Wuhan-Hu-1 strain)**
Accession number: NC_045512.
**SARS-CoV-2 Alpha**
Accession number: EPI_ISL_600093.
**SARS-CoV-2 Beta**
D80A, D251G, 242–243del, K417N, E484K, N501Y, D614G, A701V.
**SARS-CoV-2 Delta**
T19R, G142D, 157–158 del, L452R, T478K, D614G, P681R, D950N.
**SARS-CoV-2 Eta**
Q52R, E484K, Q677H, F888L.
**SARS-CoV-2 Epsilon**
L5 del, P9 del, S13I, W152C, L452R.
**SARS-CoV-2 Mu**
Accession number: EPI_ISL_4659819.
**SARS-CoV-2 Iota**
Accession number: EPI_ISL_1708781.
**SARS-CoV-2 Lambda**
Accession number: EPI_ISL_1321479.
**SARS-CoV-2 Kappa**
Accession number: EPI_ISL_2741391.
**SARS-CoV-2 C.1.2**
P9L, C136F, 144-145del, R190S, D215G.
**SARS-CoV-2 Omicron BA.1**
A67V, 69–70del, T95I, G142D, 143–145del, N211I, 212del, 215EPEins, G339D, S371L, S373P, S375F, K417N, N440K, G446S, S477N, T478K, E484A, Q493R, G496S, Q498R, N501Y, Y505H, T547K, D614G, H655Y, N679K, P681H, N764K, D796Y, N856K, Q954H, N969K, L981F.
**SARS-CoV-2 Omicron BA.2**
Accession number: EPI_ISL_9652748.
**SARS-CoV-2 Omicron BA.4**
Accession number: EPI_ISL_11542550.
**SARS-CoV-2 Omicron BA.2.75**
Accession number: EPI_ISL_13502576.
**SARS-CoV-2 Omicron BQ.1.1**
Accession number: EPI_ISL_14818139.
**SARS-CoV-2 Omicron XBB.1**
Accession number: EPI_ISL_14917761.
**SARS-CoV-2 Omicron EG.5.1**
Accession number: EPI_ISL_17854292.
**SARS-CoV-2 Omicron BA.2.86**
Accession number: EPI_ISL_18110065.
Additional mutations were constructed by the site-directed mutagenesis based on above SARS-CoV-2 spike genes using the Mut Express II Fast Mutagenesis Kit V2 (Vazyme Biotech).

### SARS-CoV-2 pseudovirus infection enhancement or neutralization assay

The continuously diluted mAb starting from 50 µg/mL was co-incubated with an equal-volume of pseudovirus at 37°C for 1 h and then added to the prepared 96-well white plate containing HEK293T-hACE2 cells with DEAE-dextran (1:1000). After 48 h post-incubation at 37 °C with 5% CO_2_, the culture medium was removed and 100 μL bright-Lite Luciferase reagent (Vazyme Biotech) was added to each well, then the plate was shaked at room temperature for 2 min. The Luciferase activity was measured by using Varioskan TM LUX Multimode Microplate Reader. EC_50_ were determined by fitting data with log (agonist) vs. normalized response - Variable slope (four parameters) mode using GraphPad Prism 8.0 software.

### Authentic SARS-CoV-2 Delta variant infection assay

Authentic SARS-CoV-2 Delta variant virus (a clinical isolate) focus forming assay (FFA) to evaluate the effect of antibodies on infection was performed in a certified Biosafety Level 3 (BSL-3) laboratory. Serial dilutions of testing mAbs were mixed with 50 μL of SARS-CoV-2 (100 focus forming units, FFU) in 96-well microwell plates and incubated at 37 °C for 1 h. Mixtures were then transferred to 96-well plates seeded with Vero E6 cells for adsorption at 37 °C for 1 h. Inoculums were then removed before adding the overlay media (100 μL MEM containing 1.6% Carboxymethylcellulose). The plates were then incubated at 37 °C for 24 h. Overlays were removed, and cells were fixed with 4% paraformaldehyde solution for 30 min, permeabilized with Perm/Wash buffer (BD Biosciences) containing 0.1% Triton X-100 for 10 min. Cells were incubated with rabbit anti-SARS-CoV-2 NP IgG (1:1000 dilution; Sino Biological Inc.) for 1 h at room temperature before adding HRP-conjugated goat anti-rabbit IgG (H + L) antibody (TransGen Biotech). The reactions were developed with KPL TrueBlue Peroxidase substrates (Seracare Life Sciences Inc.). The numbers of SARS-CoV-2 foci were quantified using an ELISpot reader (Cellular Technology Ltd.).

### Fragment of antigen binding F(ab’)_2_ and Fab generation

ConD-854 Fab was obtained by fragmentation of ConD-854 IgG. The full length of IgG was diluted to 1 mg/mL, then L-Cysteine hydrochloride (final concentration 20 mM, Sigma-Aldrich), EDTA (final concentration 20 mM, Invitrogen), and papine (final concentration 1.25 mg/mL, Sigma-Aldrich) were added and incubated at 37°C for 12–14 h. The reaction was stopped by adding 0.5 M Iodoacetamide (Sigma Aldrich). Fab was purified by using protein A affinity chromatography to separate the fragment crystallizable (Fc) and redundant undigested IgG. To generate F(ab’)_2_ format Abs, the full-length IgG was digested using pepsin protease with the Pierce F(ab’)_2_ Preparation Kit (Thermo Fisher) according to the manufacturer’s instructions. The purified F(ab’)_2_ and Fab fragment were further validated by SDS-PAGE, and the gel was stained with Coomassie brilliant blue to visualize the protein bands. Additionally, to confirm the purity of purified F(ab’)_2_ and Fab fragment samples by determining residual full-length IgG, we used a PE-conjugated anti-human IgG Fc secondary antibody (eBioscience) and performed residual IgG detection via flow cytometry to assess its binding to the spike protein of the Delta variant on the cell surface.

### Cell-surface NTD or spike protein binding

To evaluate antibody binding, HEK293T cells were transfected with different plasmid constructs using EZ Trans (Life-ilab). The full-length SARS-CoV-2 spike protein (and its mutants) were co-transfected with the pCAGGS-GFP vector at a 1:1 ratio, whereas the Flag-NTD-TM plasmid was transfected alone. At 36 h post-transfection, cells were harvested and stained with LIVE/DEAD Fixable Green Dead Cell Stain kit (Invitrogen) for viability. For cells expressing the full-length spike, binding of the NTD mAb (10 µg/mL) was assessed. After incubation and washing, Alexa Fluor 647 goat anti-human IgG (H + L) antibody (Invitrogen) was used as the secondary antibody for detection, and binding was quantified by the MFI of the NTD mAb on GFP-positive cells. For cells expressing Flag-NTD-TM, the protein was simultaneously incubated with an PE anti-Flag antibody (Biolegend) and the NTD mAb (each at 10 µg/mL). After washing, APC-conjugated anti-human IgG Fc was used for NTD mAb detection. The binding of the NTD mAb was specifically quantified by its MFI on Flag-positive cells. All samples were analyzed on a BD FACSymphony A3 Cell Analyzer, and data were were processed using FlowJo software.

### ACE2 binding enhancement determined by flow cytometry

The plasmid encoding spike with T2A self-cleaving peptide fused to the GFP protein was transiently transfected into HEK293T cells (ATCC) using EZ Trans (Life-ilab) in Opti-MEM transfection medium. After 6 h of incubation at 37 °C with 5% CO_2_, the cells were supplemented with DMEM containing 10% of FBS. After 36 or 48 h, the cells were harvested and stained with the LIVE/DEAD Fixable Dead Cell Stain Kit (Invitrogen) to exclude dead cells and were incubated with 3 μg/mL of His-tagged recombinant ACE2 ECD and the ConD-854 antibody at a starting concentration of 10 μg/mL with serial 3-fold dilutions and incubated at 4°C for 30 min. Afterwards, APC anti-6 × His tag antibody (Abcam) and mouse anti-human IgG Fab secondary antibody, PE (Thermo Fisher Scientific) were added to the cells and incubated at 4°C for 30 min. Finally, the cells were resuspended, and binding of antibody was quantified by BD FACSymphony A3 Cell Analyzer (BD Biosciences). The amounts of mAb and ACE2 bound to the spike protein on GFP-positive cells were determined by the MFI in the PE and APC channels, respectively, analyzed using FlowJo.

### H014 binding enhancement determined by flow cytometry

H014 mAb was labeled with biotin using the EZ-Link Sulfo NHS-SS Biotinylation Kit (Thermo Scientific) following the manufacturer’s recommendations. The plasmid encoding spike with T2A self-cleaving peptide fused to the GFP protein was transiently transfected into HEK293T cells (ATCC) using EZ Trans (Life-ilab) in Opti-MEM transfection medium. After 6 h of incubation at 37 °C with 5% CO_2_, the cells were supplemented with DMEM containing 10% of FBS. After 36 or 48 h, the cells were harvested and stained with the LIVE/DEAD Fixable Dead Cell Stain Kit (Invitrogen) to exclude dead cells and were incubated with 3 μg/mL of biotinylated H014 mAb and the ConD-854 mAb at a starting concentration of 10 μg/mL with serial 3-fold dilutions and incubated at 4°C for 30 min. Afterwards, APC streptavidin (Biolegend) was added to the cells and incubated at 4°C for 30 min. Finally, the cells were resuspended, and binding of antibody was quantified by BD FACSymphony A3 Cell Analyzer (BD Biosciences). The amounts of H014 mAb bound to the spike protein on GFP-positive cells were determined by the MFI in the APC channel analyzed using FlowJo.

### Enzyme linked immunosorbent assay (ELISA) for determination of antibody binding activities

The antigen protein was coated onto 96-well plates and incubated at 4°C overnight. The plates were washed with PBST and blocked with 5% skim milk and 2% bovine serum albumin (BSA) in PBS at RT for 1 h. mAbs were added into wells and incubated at 37°C for 1 h. After washing, HRP-conjugated goat anti-human IgG antibodies (ZSGB-BIO) were added and incubated at 37°C for 1 h. Finally, the TMB substrate (Sangon Biotech) was added and incubated at RT for 20 min, and the reaction was stopped by 2 M H_2_SO_4_. Absorbance was measured at 450 nm.

### Epitope mapping by antibody-competition ELISA

ELISA plates were coated with prototype spike trimer (2 μg/mL) in PBS at 4°C overnight, washed with PBST, and blocked with 5% skim milk and 2% BSA in PBS for 1 h at RT. HRP conjugated (Abcam) ConD-854 was added with serially diluted competitor antibodies and incubated at 37°C for 1 h. TMB substrate (Sangon Biotech) was added, incubated at RT for 20 min, and the reaction stopped with 2 M H_2_SO_4_. Absorbance was measured at 450 nm. VRC01 (HIV-1-specific mAb) served as a negative control, showing no competition with SARS-CoV-2 spike-specific mAbs. The percentage of competition was calculated by the formula: (1 − OD_450 nm_ of tested mAb/OD_450 nm_ of VRC01 control)× 100%.

### Cryo-EM sample preparation and data acquisition

The Delta spike-ConD-854 IgG complex was concentrated to ~1.5 mg/mL. Aliquots (3.3 μL) of the complex were applied onto glow-discharged holey carbon grids (Quantifoil Au R1.2/1.3) using a Vitrobot (Mark IV, Thermo Fisher Scientific), with blotting time of 3.0 s or 3.5 s, followed by flash-frozen in liquid ethane cooled by liquid nitrogen. The prepared grids were transferred to a Titan Krios transmission electron microscope operating at 300 kV, equipped with a Gatan K3 detector and a GIF Quantum energy filter. Movie stacks were automatically collected using EPU software (Thermo Fisher Scientific) in super-resolution mode at a nominal magnification of 81,000 × , with a 20eV slit width of on the energy filter and a defocus range of -1.4 µm to -1.8 µm. Each stack was exposed for 2.56 s (0.08 s per frame), resulting in 32 frames per stack. The total dose was approximately 50 e^-^/Å^2^ per stack. Stacks were motion-corrected using MotionCor2 [57] and binned 2 × , yielding a pixel size of 1.087 Å. Dose weighting was performed [58], and defocus values were estimated with Gctf [59].

### Data processing

A total of 1,396 micrograph stacks were acquired. Particles were automatically picked from manually selected micrographs using Relion 3.0.6 [60–63]. After several rounds of 2D classification with Relion, high-quality particles were selected and subject to multiple cycle of heterogeneous refinement without symmetry constraints in cryoSPARC [64]. The good particles were further processed via Non-uniform Refinement, yielding a 3D reconstruction of the entire structure. For interface between the spike trimer NTD and ConD-854, the dataset underwent additional refinement with an adapted mask to improve map quality. The datasets of three NTD-ConD-854 Fab sub-complexes were combined and subject to focused refinement in Relion, resulting in a higher-quality 3D reconstruction of the NTD-ConD-854 Fab interface at a resolution of 3.4Å.

The resolution was estimated using the gold-standard Fourier shell correlation (FSC) 0.143 criterion [65], with high-resolution noise substitution applied [66]. Details of data collection and processing are provided in S7 and S8 Figs, and S1 Table.

### Model building and structure refinement

The predicted atomic model of ConD-854 was obtained using alphafold2.1 [67]. For model building of the spike-ConD-854 complex, the atomic model of the spike trimer bound to 4A8 (PDB: 7C2L) was used as a template. This template was subjected to molecular dynamics flexible fitting (MDFF) [68] into the full cryo-EM map of the complex and the focused-refined cryo-EM map of the NTD-ConD-854 Fab sub-complex, respectively. During model building, each residue was manually checked with consideration of its chemical properties. Segments with missing corresponding densities were not modeled. Structural refinement was performed in Phenix [69] using secondary structure and geometry restraints to prevent overfitting. To monitor potential overfitting, the model was refined against one of the two independent half maps from the gold-standard 3D refinement approach and validate against the other map. Statistics for data collection, 3D reconstruction and model building are summarized in S1 Table.

### Definition for dr, θr, and dn

The definitions of dr, θr, and dn are based on the previous method [48]. Briefly, Atomic models for Delta spikes from that do not bind to any antibodies or ACE2 (PDB: 7W92, 7W94, 7V7O, 7V7P, 7V7Q, 7V7R, 7V7S, 7V7T, 7V7U, 7V7V, 7TEY, 7V7N, 7SBK, 7SBL, 8HRI, 8HRJ, 9CRH, 7TOU, 7TOV, 7TP7, 7TP8, 7TP9, 7TPF, 7SBP) and prototype spike that bind to the infection-enhanced antibody DH1052 (PDB: 7LAB) were acquired from PDB database. RBD, the central axis, the rotation axis, and NTD correspond to the centroids of the domains spanning residues 334–453 + 492–527, 976–996, 541–586, and 27–303, respectively. dr, θr, and dn are all distances or angles between centroids: dr refers to the distance between RBD and the central axis, while θr denotes the angle formed by RBD-rotation axis-central axis. These two parameters are used to characterize the swinging state of RBD. Meanwhile, dn is the distance between NTD and the central axis, which serves to characterize the swinging state of NTD. The centroids of the domains were defined using the “define centroid” command in ChimeraX; distance and angle measurements were performed using the “distance” and “angle” commands in the same software.

### Importance

We identified an NIEA induced by the primary infection of SARS-CoV-2 Delta variant, named ConD-854, whose NTD-binding mode is distinct from that of previously reported prototype-NIEAs, featuring a near-perpendicular orientation of heavy-light chain when binding to similar epitopes relative to prototype-NIEAs.

## Supporting information

S1 Fig Biochemical and functional characterization of ConD-854. **(A)** The infection-enhancing activity of ConD-854 against the SARS-CoV-2 Delta variant was evaluated using a pseudovirus-based assay in Vero E6 and 293T-ACE2 cells, with C144 (a neutralizing RBD-targeting antibody) and VRC01 (an HIV-1-specific mAb) serving as controls. **(B)** Binding assay determined by ELISA evaluating the binding capability of COV2–2490 to S1, NTD, and RBD of SARS-CoV-2 Delta spike. mAbs were tested at an initial concentration of 10 μg/mL and serially diluted by 5-fold. (A, B) Experiments were performed with two biological replicates (each in technical duplicate), yielding consistent results. Data from one representative experiment are presented. **(C)** Analysis of purified ConD-854 IgG, F(ab’)_2_ and Fab under non-reducing (Native Gel Sample Loading Buffer, 5 × , Beyotime) and reducing (6 × SDS-PAGE loading buffer, with DTT, TransGen) conditions. SDS-PAGE was performed on 10% gel, with both denatured and non-denatured protein samples loaded for analysis. The gel was visualized via standard Coomassie brilliant blue staining, and data shown is representative of two independent experiments. **(D)** Flow cytometric analysis of ConD-854 IgG, F(ab’)_2_ and Fab binding to the cell-surface expressed Delta spike with a PE-conjugated goat anti-human IgG Fc secondary antibody. VRC01 is an HIV-1 specific mAb, used as a negative control here. **(E)** Binding capacity of ConD-854 in the formats of IgG, F(ab’)_2_, and Fab to SARS-CoV-2 Delta spike trimer. Data presented here are means of three independent experiments with technical duplicate and error bars represent ± SD.
(TIF)

S2 Fig Enhanced ACE2 binding to whole spike protein on cell surface by bivalent ConD-854. **(A)** Gating strategies for analyzing enhanced binding of ACE2 to the spike protein by ConD-854. **(B)** Whole spike transfectants (prototype, D614G, Delta) were stained with full-length IgG-, F(ab’)_2_- and Fab- form of ConD-854 individually, as well as control antibodies (C135, CV1, VRC01). Bound antibodies were detected by PE labeled anti-human IgG Fab specific antibodies. **(C)** Enhanced binding of ACE2 to the spike protein by IgG-, F(ab’)_2_- and Fab- form of ConD-854 determined by flow cytometry. Negative controls included the Class 3 anti-SARS-CoV-2 RBD mAb C135 (sterically hinders ACE2), the anti-SARS-CoV-2 NTD mAb CV1, and the HIV-1-specific mAb VRC01. The representative data of two independent experiments are shown.
(TIF)

S3 Fig *In vitro* analysis of the effect of NIEA ConD-854 on the RBD-targeting nAbs in blocking SARS-CoV-2 pseudovirus infection. **(A-C)** Effects of ConD-854 combined with RBD-targeting nAbs on SARS-CoV-2 pseudovirus infection in HEK-293T-ACE2 cells. ConD-854 was tested either alone (A) or in combination with nAbs H014 or C144 at fixed ratios (1:1 or 10:1) (B), or at a fixed concentration of ConD-854 with serially diluted nAbs H014 or C144 (C). Experiments were performed with two biological replicates (each in technical duplicate), yielding consistent results. Data from one representative experiment are presented.
(TIF)

S4 Fig The cross-binding capability of ConD-854 to spike proteins of SARS-CoV-2 variants. ELISA assay detected ConD-854 and the reported NIEAs (COV2–2490, COV2–2369, DH1052 and 8D2) binding to spike trimers of several SARS-CoV-2 variants. CV1 is a SARS-CoV-2 NTD-targeting neutralizing antibody and serves as a positive control. VRC01 is an HIV-1 specific mAb and serves as a negative control here. The representative data of two independent experiments are shown.
(TIF)

S5 Fig Identification of key amino acid residues the NTD recognized by ConD-854 using site-directed mutagenesis. **(A)** Sequence alignment and possible mutations in the NTD conferring escape from ConD-854. Mutations in the NTD region of several representative SARS-CoV-2 variants, compared to the prototype strain, are shown (light yellow). The sites with mutations exclusively present in variants not recognized by ConD-854 are highlighted in orange and these sites were selected as potential key sites for further verification. The SARS-CoV-2 variants of which spikes were recognized by ConD-854 are highlighted in green. In contrast, the variants not recognized by ConD-854 are highlighted in purple. − : deletion, in: insertion. **(B)** Fold-change in infection enhancement by ConD-854 (concentration = 10 μg/mL) against SARS-CoV-2 Delta single-point mutant strains, as detected by pseudovirus infection enhancement assays. The data in (B) are from three independent experiments and presented as mean ± SD. Statistical significance was performed using an unpaired t-test (*p < 0.05, **p < 0.01). n.s.: not significant.
(TIF)

S6 Fig ConD-854 epitope mapping by competitive ELISA. A competitive ELISA was performed to detect HRP-conjugated ConD-854 binding to the immobilized prototype spike trimer in the presence of different 5-fold serial dilution of unconjugated prototype-induced NIEAs (COV2–2490, DH1052 and 8D2) at an initial concentration of 10 μg/mL.
(TIF)

S7 Fig Cryo-EM analysis of the Delta spike-ConD-854 complex. **(A)** Gold-standard FSC curves for the Relion 3D reconstructions of the Delta spike-ConD-854 complex and NTD-ConD-854 subcomplex. **(B)** Euler angle distributions of the ternary complex in the final 3D reconstruction of whole map (left) and the local map of NTD-ConD-854 interface (right). **(C)** FSC curves for the refined model of spike-ConD-854 complex (left) and NTD-ConD-854 subcomplex (right). The black curve represents the refined model versus the summed map. The red curve shows the model refined against the first half map versus the first half map itself, while the green curve shows the model refined against the first half map versus the second half map. **(D)** Flowchart of cryo-EM data processing (details in the Data Processing section of the Methods. **(E, F)** The local resolution map of spike-ConD-854 complex (E) and NTD-ConD-854 subcomplex (F).
(TIF)

S8 Fig Representative cryo-EM density maps are shown at threshold of 7 σ. Representative density maps for residues 27–33, 189–200, and 207–220 of the Delta spike NTD, as well as residues 91–100 of the ConD-854 heavy chain and residues 36–45 of the light chain.
(TIF)

S9 Fig Angles of approach for ConD-854 and other three prototype-induced NIEAs binding to the NTD of Delta spike. NIEAs binding to a similar NTD epitope. The colored “sticks” represent lines connecting the average Cα position of each antibody’s variable region to the average Cα position of the Delta NTD domain, colored as indicated. The PDB IDs of the spike trimer complexed with DH1052, 8D2, and COV2–2490 are 7LAB, 7DZX, and 7DZY, respectively.
(TIF)

S10 Fig Graphical Abstract. ConD-854, a Delta strain induced NIEA, recognized a roughly similar region on the NTD containing the Loop211–215, but exhibits a distinct binding mode when compared with the previously reported prototype-induced NIEAs, as its heavy-light chain orientation is nearly perpendicular to that of the prototype-induced NIEAs. The graphic was created with Biorender (biorender.com).
(TIF)

S1 Table Cryo-EM data collection and refinement statistics. (XLSX)

S2 Table The germline gene analysis of ConD-854. (XLSX)

S1 File Source data. (XLSX)
