# Supplementary figures and images for: A SARS-CoV-2 variant-induced NTD-targeting antibody enhances viral infection via a distinctive binding mode
Source: PLoS Pathog. 2026 Feb 10;22(2):e1013828. doi: 10.1371/journal.ppat.1013828 (PMC12890138; doi:10.1371/journal.ppat.1013828)

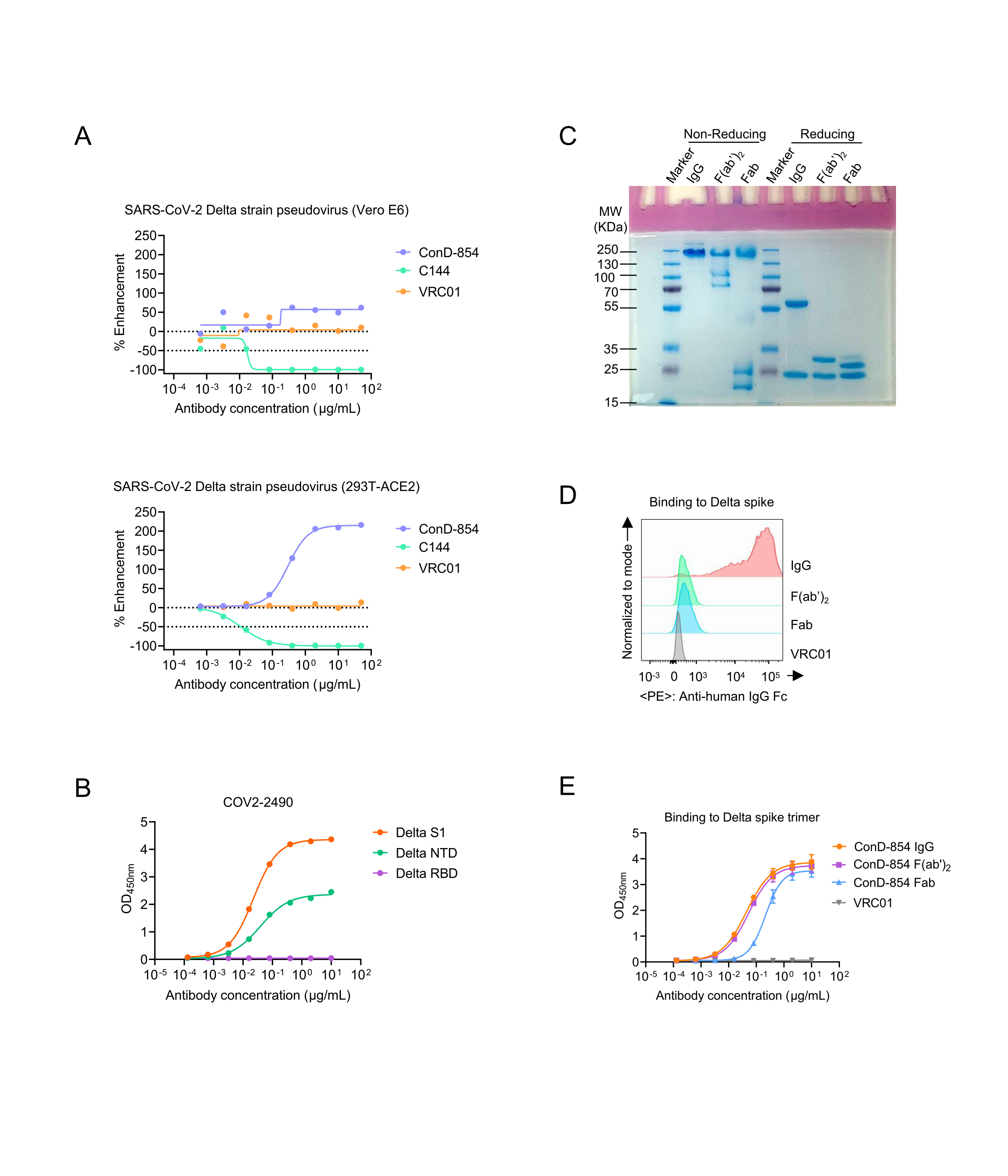

Supplement: S1 Fig — (A) The infection-enhancing activity of ConD-854 against the SARS-CoV-2 Delta variant was evaluated using a pseudovirus-based assay in Vero E6 and 293T-ACE2 cells, with C144 (a neutralizing RBD-targeting antibody) and VRC01 (an HIV-1-specific mAb) serving as controls. (B) Binding assay determined by ELISA evaluating the binding capability of COV2–2490 to S1, NTD, and RBD of SARS-CoV-2 Delta spike. mAbs were tested at an initial concentration of 10 μg/mL and serially diluted by 5-fold. (A, B) Experiments were performed with two biological replicates (each in technical duplicate), yielding consistent results. Data from one representative experiment are presented. (C) Analysis of purified ConD-854 IgG, F(ab’)2 and Fab under non-reducing (Native Gel Sample Loading Buffer, 5 × , Beyotime) and reducing (6 × SDS-PAGE loading buffer, with DTT, TransGen) conditions. SDS-PAGE was performed on 10% gel, with both denatured and non-denatured protein samples loaded for analysis. The gel was visualized via standard Coomassie brilliant blue staining, and data shown is representative of two independent experiments. (D) Flow cytometric analysis of ConD-854 IgG, F(ab’)2 and Fab binding to the cell-surface expressed Delta spike with a PE-conjugated goat anti-human IgG Fc secondary antibody. VRC01 is an HIV-1 specific mAb, used as a negative control here. (E) Binding capacity of ConD-854 in the formats of IgG, F(ab’)2, and Fab to SARS-CoV-2 Delta spike trimer. Data presented here are means of three independent experiments with technical duplicate and error bars represent ± SD. (TIF) [file ppat.1013828.s001.tif]

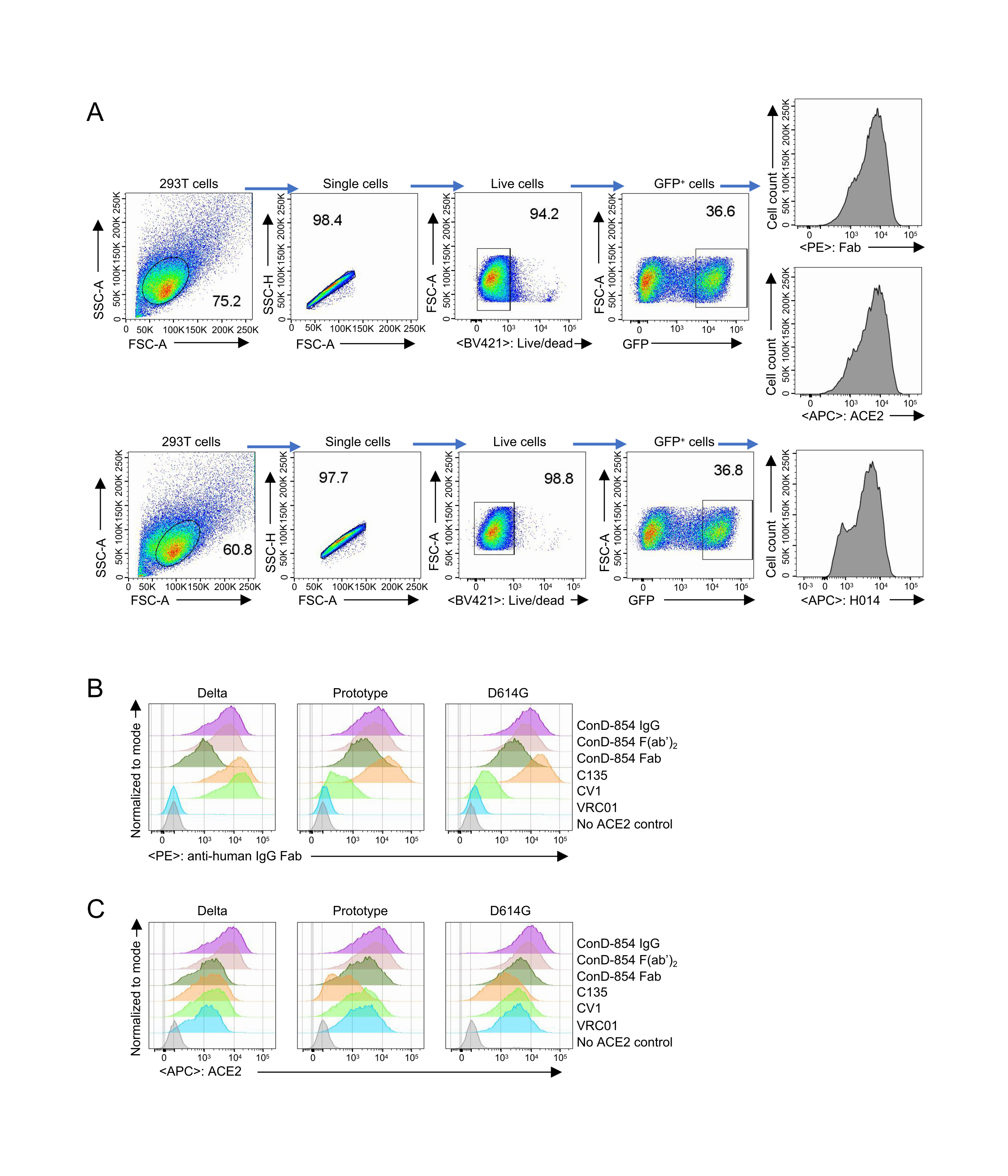

Supplement: S2 Fig — (A) Gating strategies for analyzing enhanced binding of ACE2 to the spike protein by ConD-854. (B) Whole spike transfectants (prototype, D614G, Delta) were stained with full-length IgG-, F(ab’)2- and Fab- form of ConD-854 individually, as well as control antibodies (C135, CV1, VRC01). Bound antibodies were detected by PE labeled anti-human IgG Fab specific antibodies. (C) Enhanced binding of ACE2 to the spike protein by IgG-, F(ab’)2- and Fab- form of ConD-854 determined by flow cytometry. Negative controls included the Class 3 anti-SARS-CoV-2 RBD mAb C135 (sterically hinders ACE2), the anti-SARS-CoV-2 NTD mAb CV1, and the HIV-1-specific mAb VRC01. The representative data of two independent experiments are shown. (TIF) [file ppat.1013828.s002.tif]

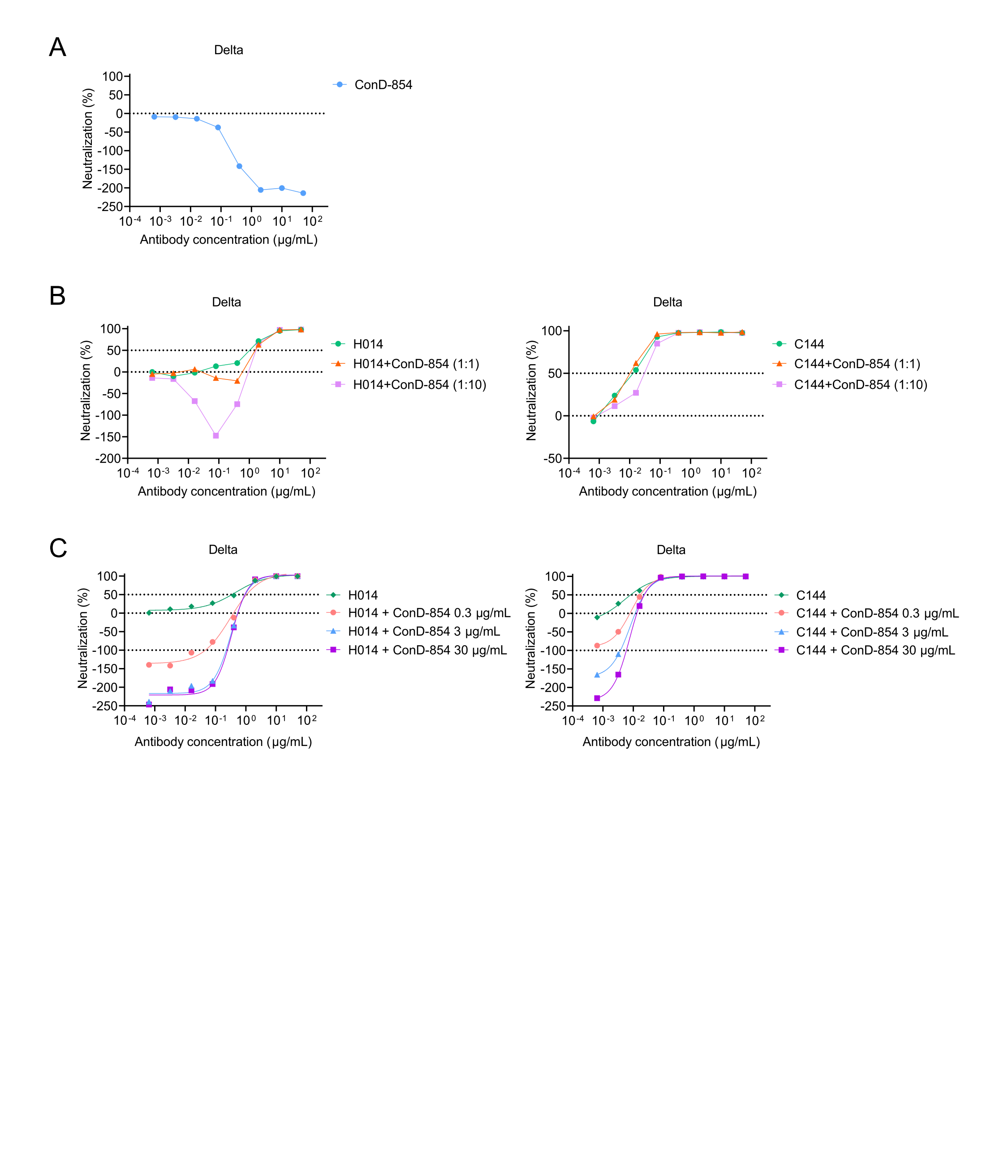

Supplement: S3 Fig — (A-C) Effects of ConD-854 combined with RBD-targeting nAbs on SARS-CoV-2 pseudovirus infection in HEK-293T-ACE2 cells. ConD-854 was tested either alone (A) or in combination with nAbs H014 or C144 at fixed ratios (1:1 or 10:1) (B), or at a fixed concentration of ConD-854 with serially diluted nAbs H014 or C144 (C). Experiments were performed with two biological replicates (each in technical duplicate), yielding consistent results. Data from one representative experiment are presented. (TIF) [file ppat.1013828.s003.tif]

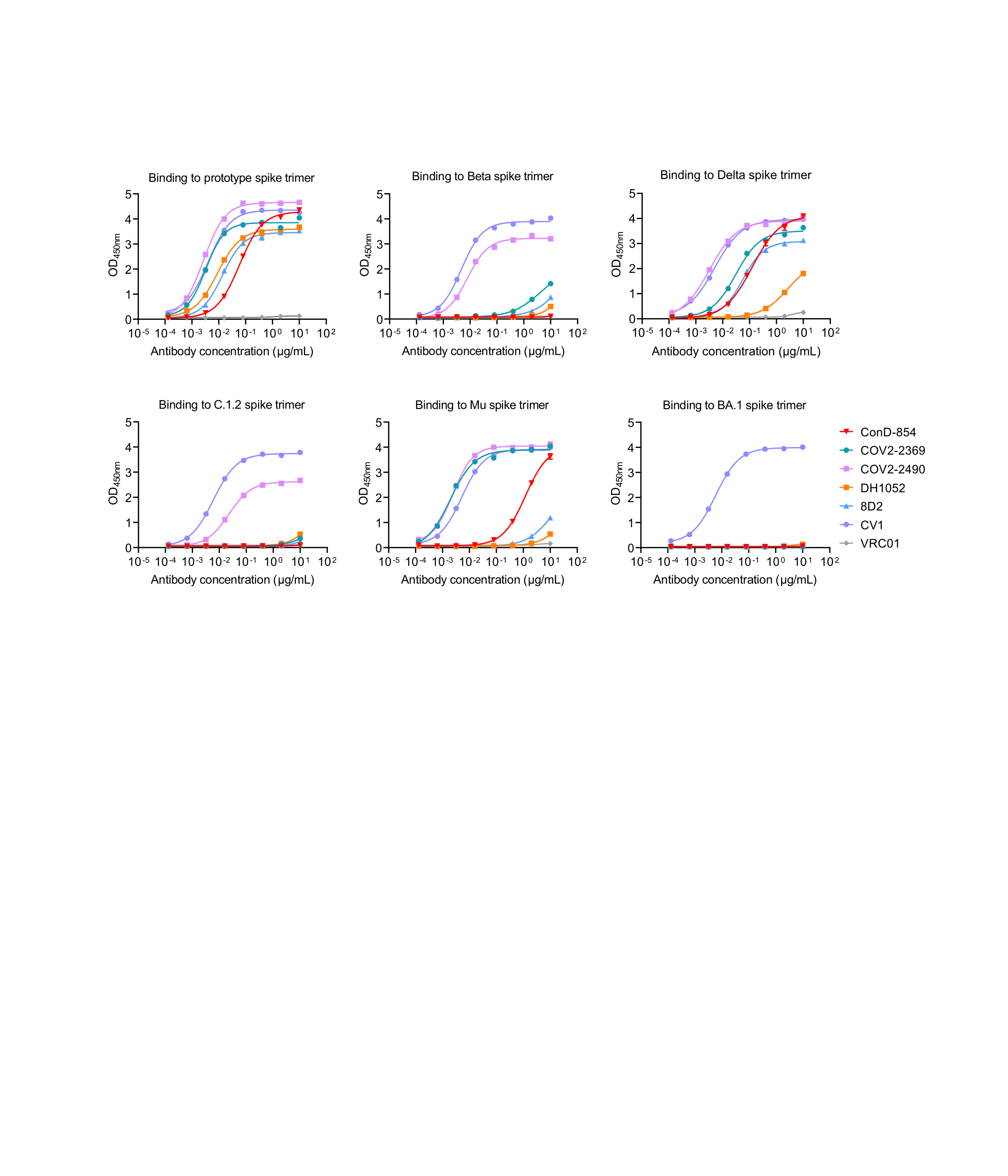

Supplement: S4 Fig — ELISA assay detected ConD-854 and the reported NIEAs (COV2–2490, COV2–2369, DH1052 and 8D2) binding to spike trimers of several SARS-CoV-2 variants. CV1 is a SARS-CoV-2 NTD-targeting neutralizing antibody and serves as a positive control. VRC01 is an HIV-1 specific mAb and serves as a negative control here. The representative data of two independent experiments are shown. (TIF) [file ppat.1013828.s004.tif]

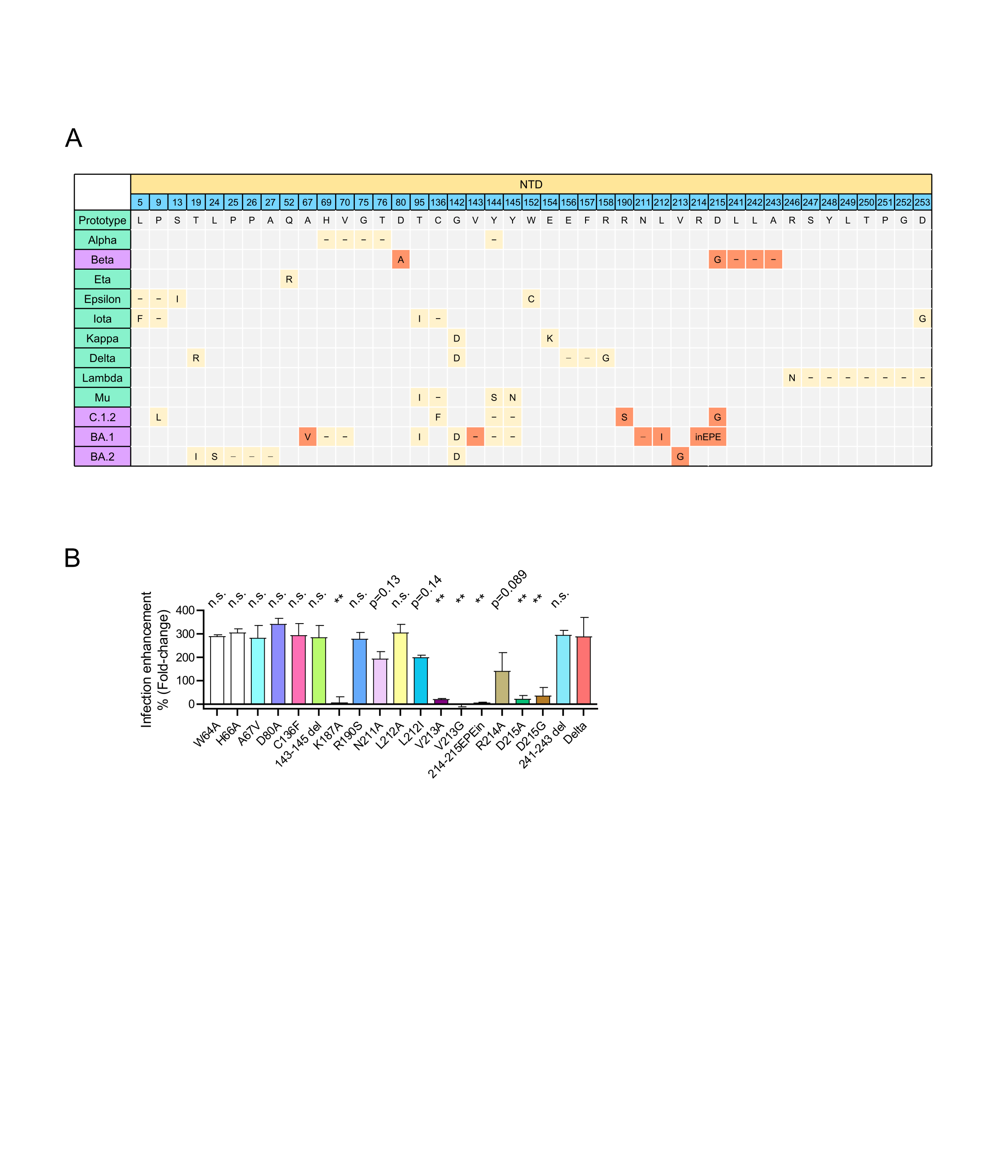

Supplement: S5 Fig — (A) Sequence alignment and possible mutations in the NTD conferring escape from ConD-854. Mutations in the NTD region of several representative SARS-CoV-2 variants, compared to the prototype strain, are shown (light yellow). The sites with mutations exclusively present in variants not recognized by ConD-854 are highlighted in orange and these sites were selected as potential key sites for further verification. The SARS-CoV-2 variants of which spikes were recognized by ConD-854 are highlighted in green. In contrast, the variants not recognized by ConD-854 are highlighted in purple. − : deletion, in: insertion. (B) Fold-change in infection enhancement by ConD-854 (concentration = 10 μg/mL) against SARS-CoV-2 Delta single-point mutant strains, as detected by pseudovirus infection enhancement assays. The data in (B) are from three independent experiments and presented as mean ± SD. Statistical significance was performed using an unpaired t-test (*p < 0.05, **p < 0.01). n.s.: not significant. (TIF) [file ppat.1013828.s005.tif]

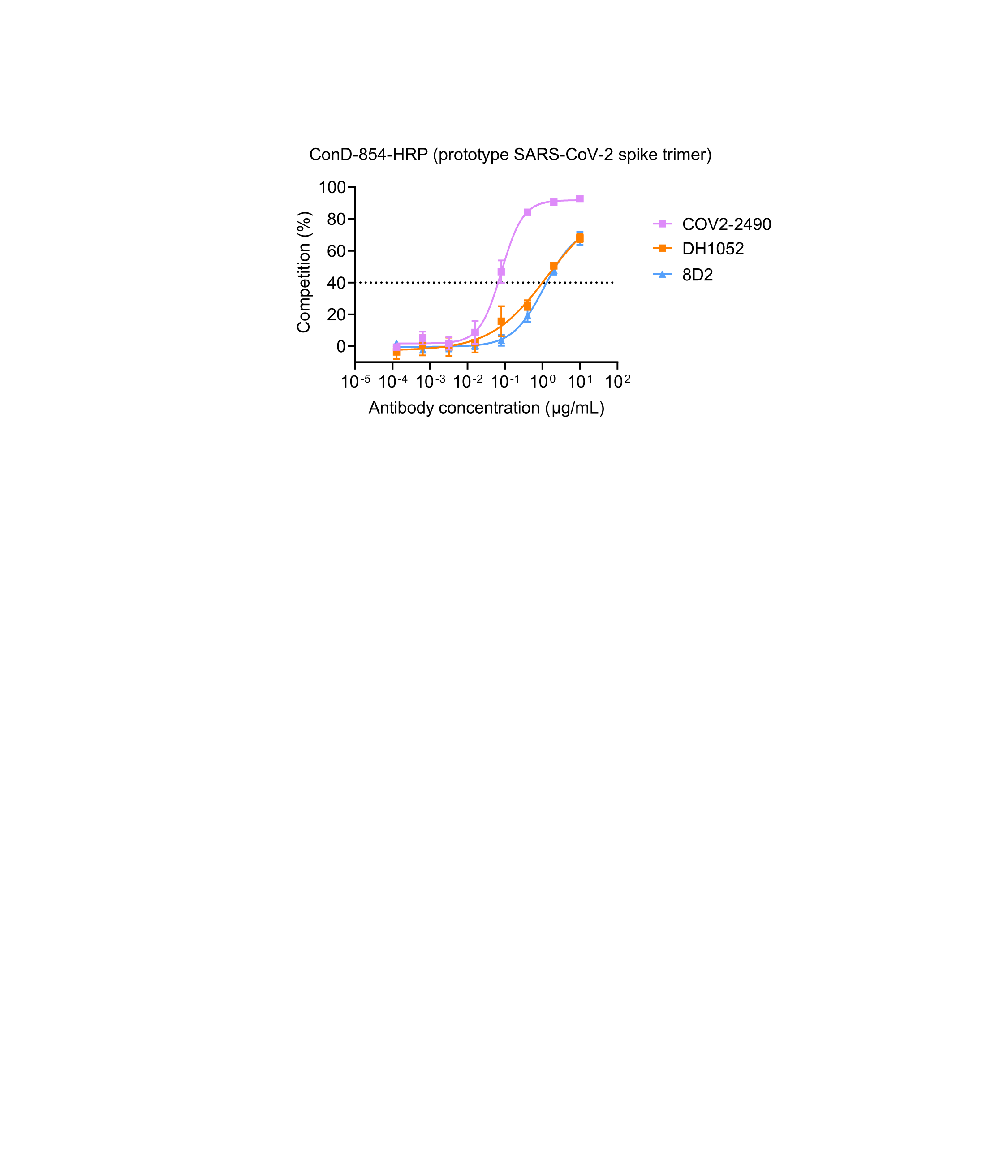

Supplement: S6 Fig — A competitive ELISA was performed to detect HRP-conjugated ConD-854 binding to the immobilized prototype spike trimer in the presence of different 5-fold serial dilution of unconjugated prototype-induced NIEAs (COV2–2490, DH1052 and 8D2) at an initial concentration of 10 μg/mL. (TIF) [file ppat.1013828.s006.tif]

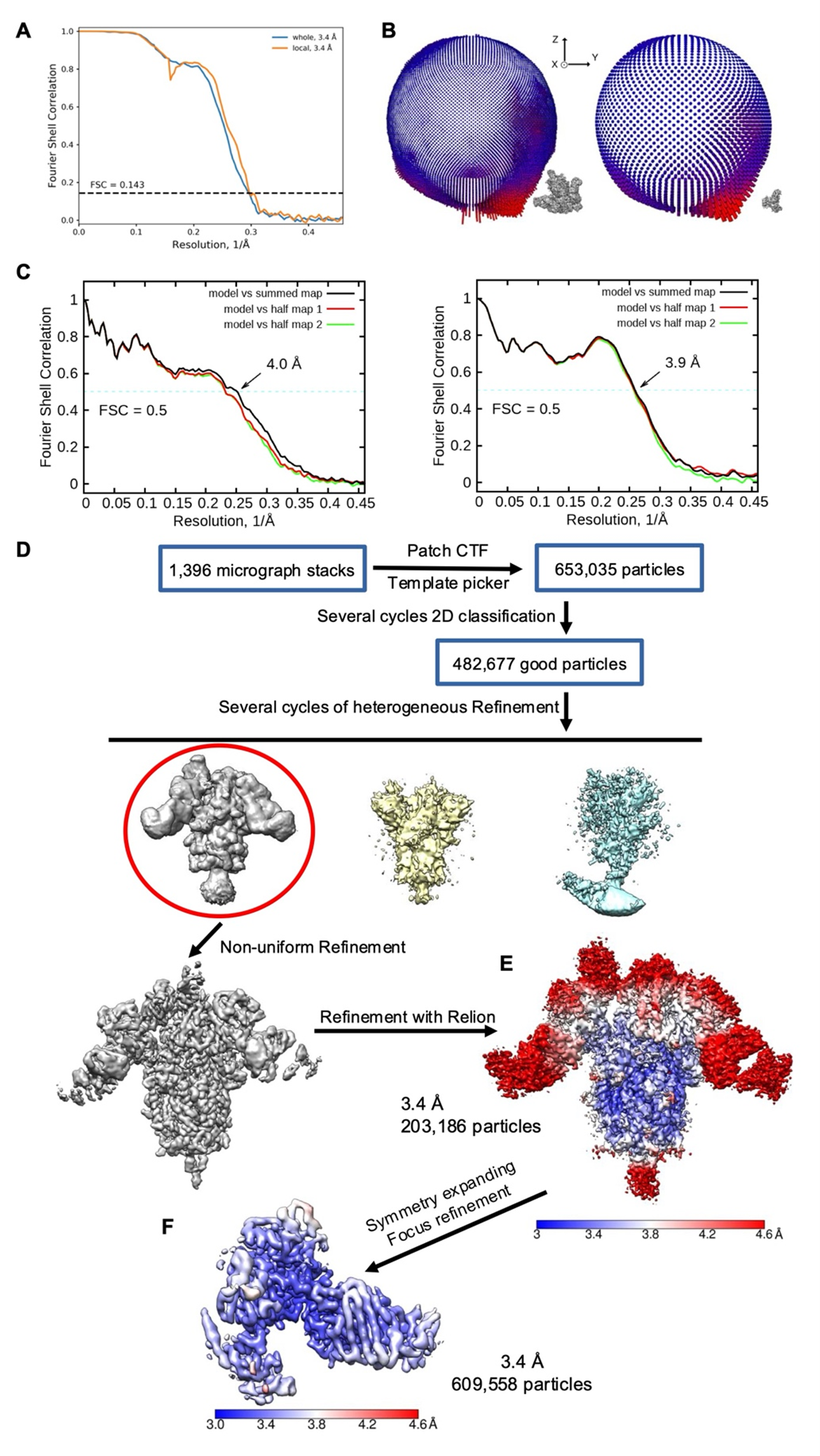

Supplement: S7 Fig — (A) Gold-standard FSC curves for the Relion 3D reconstructions of the Delta spike-ConD-854 complex and NTD-ConD-854 subcomplex. (B) Euler angle distributions of the ternary complex in the final 3D reconstruction of whole map (left) and the local map of NTD-ConD-854 interface (right). (C) FSC curves for the refined model of spike-ConD-854 complex (left) and NTD-ConD-854 subcomplex (right). The black curve represents the refined model versus the summed map. The red curve shows the model refined against the first half map versus the first half map itself, while the green curve shows the model refined against the first half map versus the second half map. (D) Flowchart of cryo-EM data processing (details in the Data Processing section of the Methods. (E, F) The local resolution map of spike-ConD-854 complex (E) and NTD-ConD-854 subcomplex (F). (TIF) [file ppat.1013828.s007.tif]

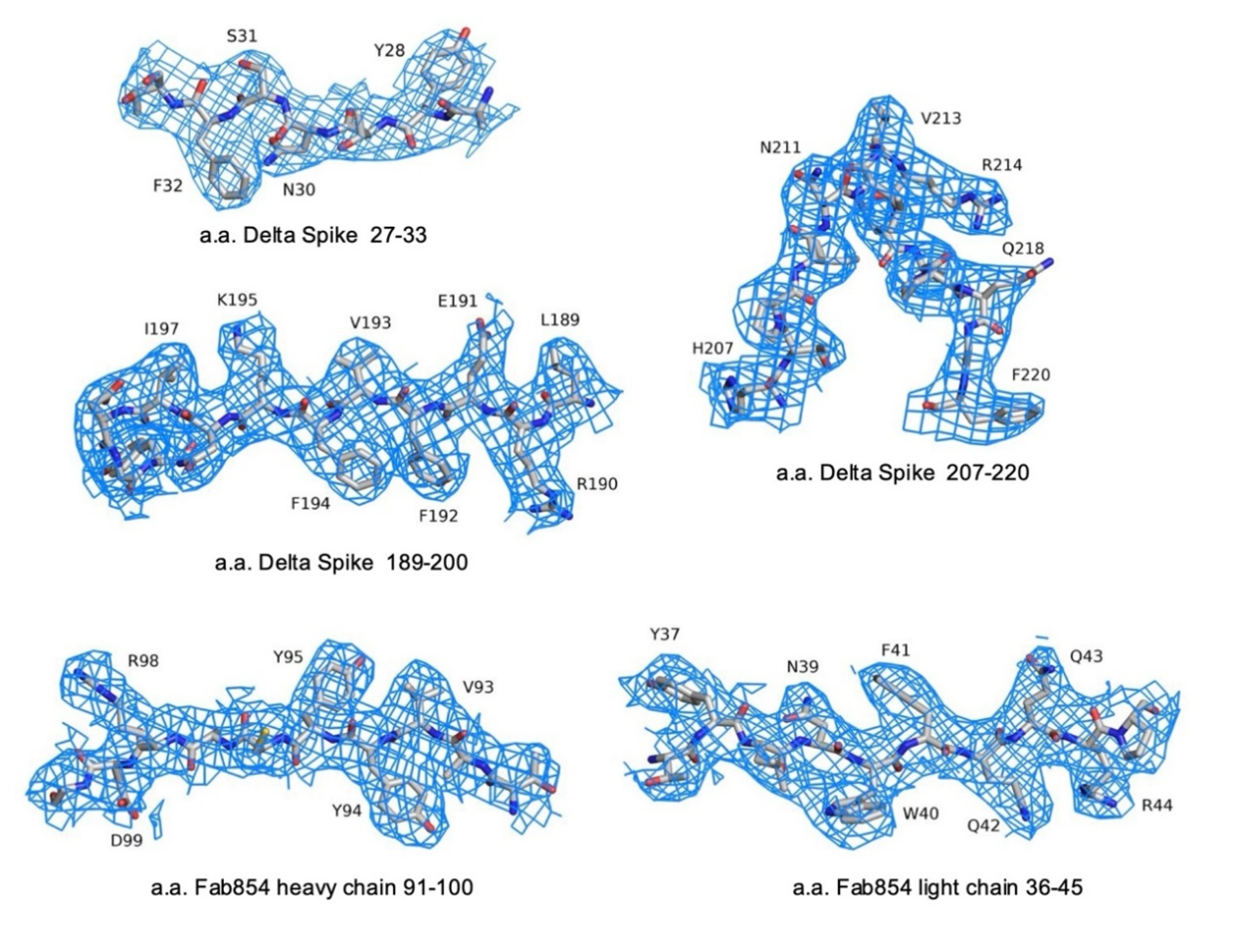

Supplement: S8 Fig — Representative density maps for residues 27–33, 189–200, and 207–220 of the Delta spike NTD, as well as residues 91–100 of the ConD-854 heavy chain and residues 36–45 of the light chain. (TIF) [file ppat.1013828.s008.tif]

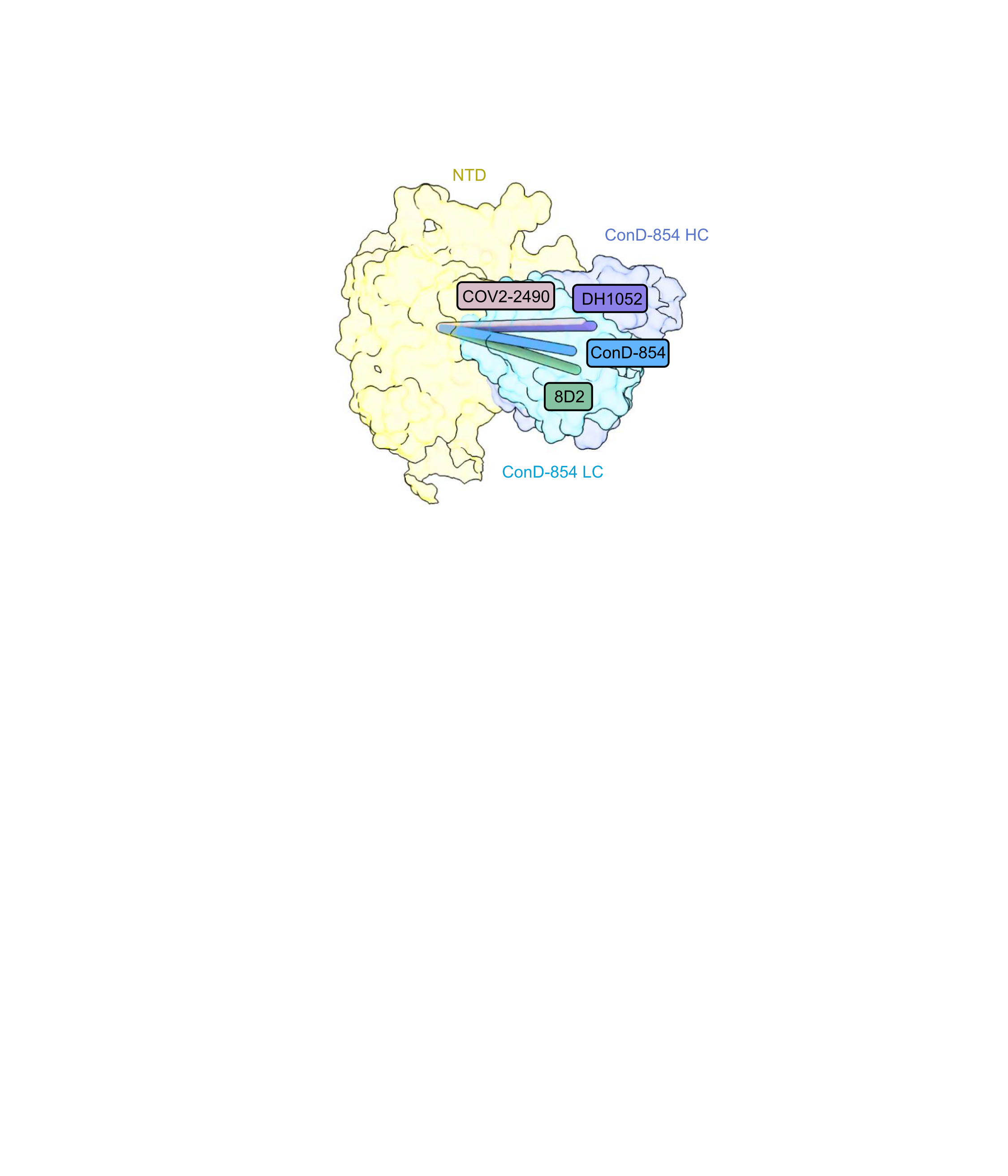

Supplement: S9 Fig — NIEAs binding to a similar NTD epitope. The colored “sticks” represent lines connecting the average Cα position of each antibody’s variable region to the average Cα position of the Delta NTD domain, colored as indicated. The PDB IDs of the spike trimer complexed with DH1052, 8D2, and COV2–2490 are 7LAB, 7DZX, and 7DZY, respectively. (TIF) [file ppat.1013828.s009.tif]

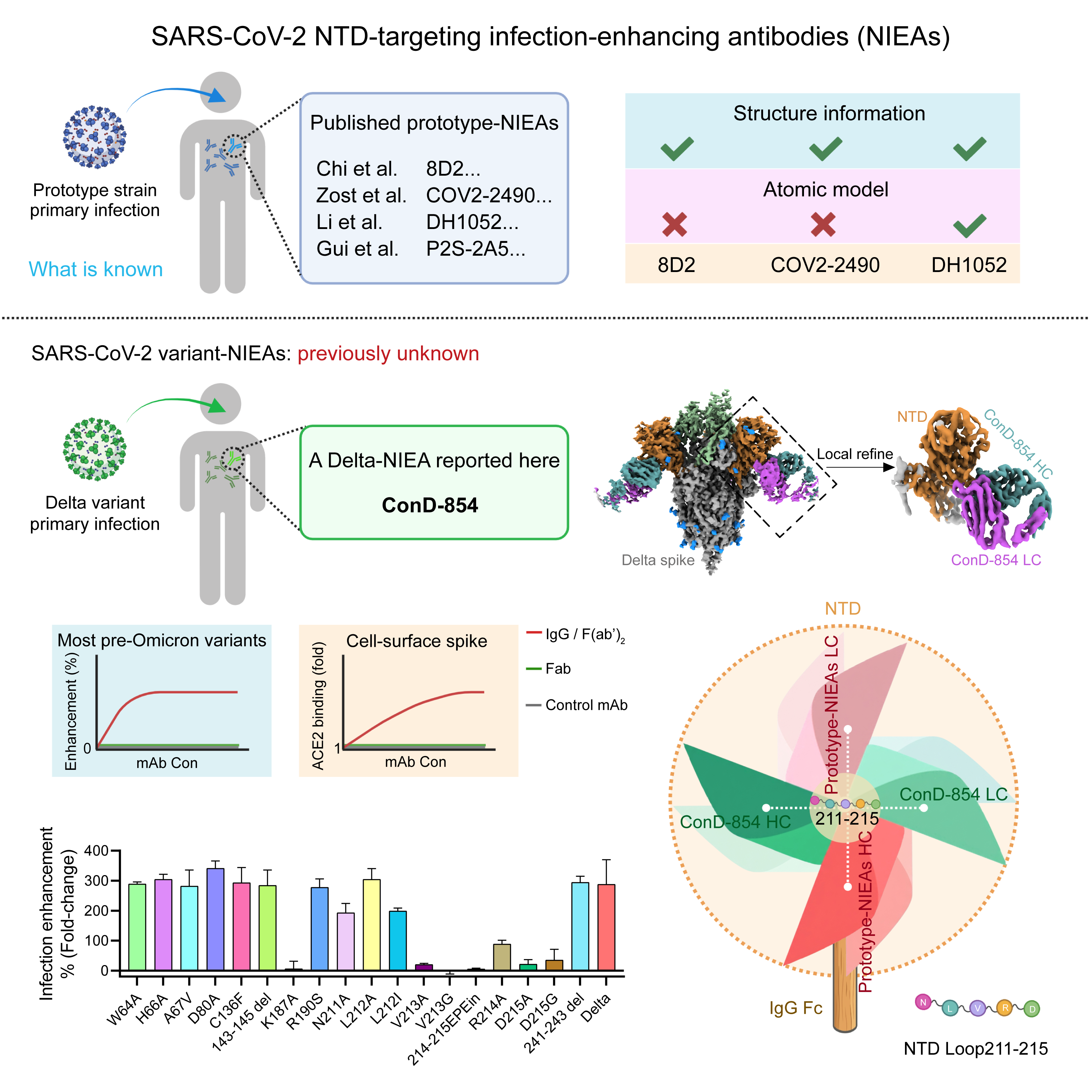

Supplement: S10 Fig — ConD-854, a Delta strain induced NIEA, recognized a roughly similar region on the NTD containing the Loop211–215, but exhibits a distinct binding mode when compared with the previously reported prototype-induced NIEAs, as its heavy-light chain orientation is nearly perpendicular to that of the prototype-induced NIEAs. The graphic was created with Biorender (biorender.com). (TIF) [file ppat.1013828.s010.tif]
